# T3SS chaperone of the CesT family is required for secretion of the anti-sigma factor BtrA in *Bordetella pertussis*

**DOI:** 10.1080/22221751.2023.2272638

**Published:** 2023-11-01

**Authors:** Jakub Držmíšek, Denisa Petráčková, Ana Dienstbier, Ivana Čurnová, Branislav Večerek

**Affiliations:** Laboratory of post-transcriptional control of gene expression, Institute of Microbiology of the Czech Academy of Sciences, Prague, Czech Republic

**Keywords:** *Bordetella pertussis*, T3SS, CesT chaperone, anti-sigma factor, biofilm

## Abstract

*Bordetella pertussis* is a Gram-negative, strictly human re-emerging respiratory pathogen and the causative agent of whooping cough. Similar to other Gram-negative pathogens, *B. pertussis* produces the type III secretion system, but its role in the pathogenesis of *B. pertussis* is enigmatic and yet to be elucidated. Here, we combined RNA-seq, LC-MS/MS, and co-immunoprecipitation techniques to identify and characterize the novel CesT family T3SS chaperone BP2265. We show that this chaperone specifically interacts with the secreted T3SS regulator BtrA and represents the first non-flagellar chaperone required for the secretion of an anti-sigma factor. In its absence, secretion but not production of BtrA and most T3SS substrates is severely impaired. It appears that the role of BtrA in regulating T3SS extends beyond its activity as an antagonist of the sigma factor BtrS. Predictions made by artificial intelligence system AlphaFold support the chaperone function of BP2265 towards BtrA and outline the structural basis for the interaction of BtrA with its target BtrS. We propose to rename BP2265 to BtcB for the *Bordetella* type III chaperone of BtrA.

In addition, the absence of the BtcB chaperone results in increased expression of numerous flagellar genes and several virulence genes. While increased production of flagellar proteins and intimin BipA translated into increased biofilm formation by the mutant, enhanced production of virulence factors resulted in increased cytotoxicity towards human macrophages. We hypothesize that these phenotypic traits result indirectly from impaired secretion of BtrA and altered activity of the BtrA/BtrS regulatory node.

## Introduction

*Bordetella pertussis* is a Gram-negative human pathogen that causes whooping cough, also known as pertussis, a highly contagious re-emerging respiratory disease. After infection, *B. pertussis* colonizes the ciliated epithelium of the human upper respiratory tract and causes inflammation, activation of immune responses and damage to host tissues [1]. To efficiently colonize the respiratory tract and evade immune response, *B. pertussis* produces a variety of virulence factors, including adhesins and toxins [1–3]. *B. pertussis* also encodes the type III secretion system (T3SS), which is an important virulence factor in many other pathogenic bacteria. In the closely related *B. bronchiseptica* the T3SS is required for persistent colonization of the lower respiratory tract of mammals [4,5]. However, compared to *B. bronchiseptica*, the role of T3SS in the pathogenesis of *B. pertussis* remains to be clarified. Previously, the T3SS was assumed to be nonfunctional in *B. pertussis* because studies on Tohama I, the highly-passaged, laboratory-adapted reference strain, failed to demonstrate secretion of T3SS substrates. Nevertheless, the T3SS was later shown to be functional in fresh clinical isolates of *B. pertussis* and in Tohama I cells exposed to iron or glutamate limitation or passaged in mice or human macrophages [6–10]. Moreover, the activity of T3SS has been reported to be increased in the presence of CO_2_ [11] or upon exposure to blood or serum [12,13]. On the other hand, after internalization by macrophages the expression of T3SS genes in intracellular *B. pertussis* and *B. bronchiseptica* cells is significantly reduced [14–16]. Furthermore, our recent studies employing highly sensitive label-free proteomic analysis using liquid chromatography coupled with tandem mass spectrometry (LC-MS/MS) proved that the laboratory-adapted strain Tohama I secretes T3SS components also under standard laboratory conditions, albeit to a much lesser extent than low-passage fresh clinical isolates [17,18].

The T3SS apparatus consists of a macromolecular injectisome that delivers effector proteins directly from the bacterial cytosol into the host cell cytosol [19]. To date, only two effector proteins have been described in bordetellae. The effector BteA, also known as BopC, is responsible for the cytotoxicity associated with T3SS in *B. bronchiseptica* and to a lesser extent in *B. pertussis* [20–23], but its mechanism of action remains unknown [24]. The other effector is the BopN protein [4,25], but this has recently been crystallized and characterized as a gatekeeper protein required for targeted translocation of the BteA effector into host cells [26]. The T3SS genes are organized in two adjacent loci: *bsc*, encoding the T3SS apparatus, and *btr*, encoding regulatory proteins [27–29], while the *bteA* effector gene is expressed from a distant locus. Expression of T3SS genes in *B. pertussis* is controlled by the two-component system BvgAS, which regulates hundreds of genes, including the extracytoplasmic function sigma factor BtrS encoded in the *btr* locus [30]. BtrS in turn activates the expression of genes within the *btr* and *bsc* loci, although the expression of some genes within these loci is not dependent on BtrS [28,30]. The activity of BtrS is inhibited by its cognate secreted antagonist BtrA (also called BspR), and in *B. pertussis*, both factors form a regulatory mechanism that couples the expression and function of T3SS [29,31]. In *B. bronchiseptica*, however, the BtrA regulon is more complex, controlling the expression of nearly 300 genes. More than 80 genes, including toxins and adhesins, are activated and over 200 genes, including flagellar genes and the T3SS operon, are repressed by BtrA [29]. Another level of regulation is added by the RNA chaperone Hfq, an important post-transcriptional regulator of Gram-negative bacteria, that has been shown to be required for the expression and functionality of the T3SS in *B. pertussis* [8,17].

Our recent study revealed that both direct exposure to blood and passage of cells from agar plates to liquid medium had a positive effect on the expression profiles of the T3SS locus in *B. pertussis* [13]. However, the response to blood exposure was not uniform, and some genes did not respond to either blood exposure or passage. Only one gene, *BP2265*, was downregulated upon transfer from solid to liquid medium. This gene was previously referred to as *orf1* because its affiliation with the T3SS was not clear [27] and its potential role in T3SS functionality was long overlooked. According to the NCBI database, the BP2265 protein shares properties with the chaperone CesT (InterPro family IPR010261). In enteropathogenic *Escherichia coli* (EPEC) strains, CesT was shown to provide a chaperone function for the translocated intimin receptor (Tir) [32,33]. Furthermore, after translocation, liberated cytosolic CesT interacts with the global translation inhibitor CsrA and antagonizes its activity [34,35].

In this study we analysed the global regulon of the BP2265 chaperone and its requirement for T3SS functionality using multiple approaches combining omics techniques, immunoproteomics, and *in silico* analyses based on artificial intelligence systems. Our data suggest that BP2265 is a cognate chaperone of the secreted anti-sigma factor BtrA and demonstrate that cells lacking the *BP2265* gene display several phenotypic traits such as impaired secretion of T3SS substrates, increased production of biofilm and enhanced cytotoxicity.

## Materials and methods

### Bacterial strains and growth conditions

*Bordetella pertussis* and *B. bronchiseptica* strains listed in Supplementary Table 1 were grown on Bordet Gengou agar (BGA, Difco) supplemented with 15% defibrinated sheep blood at 37 °C. For planktonic cultures, bacteria were gown in Stainer-Scholte medium (SSM) supplemented with 0.1% cyclodextrin and 0.5% casamino acids at 37 °C. To harvest samples for transcriptomic and proteomic analyses, three independent cultivations of B1917 strain and its isogenic Δ*BP2265* mutant were performed to collect three biological replicates. Cultures were grown overnight in SSM to mid exponential phase of growth (OD_600_ ≈ 1.3-1.6). Next, cells were pelleted by centrifugation (10,000 *g*, 4 °C, 10 min) for RNA and protein isolation. For secretome analysis, culture supernatants were filtered through 0.22 μm filters and precipitated.

### Construction of the ΔBP2265 deletion mutant and BP2265-triple FLAG tag fusion

The deletions were introduced into the chromosome of the *B. pertussis* B1917 strain as described previously [36]. To construct the Δ*BP2265* deletion mutant, two DNA fragments flanking the corresponding gene were amplified from the upstream region (which ends with initiation TTG codon of the *BP2265* gene and the *Nhe*I site) and the downstream region (which begins with the *Nhe*I site and the TAG stop codon of the *BP2265* gene). The resulting PCR products were ligated via a *Nhe*I site, and the ligation mixture was used as a template to generate a PCR product containing the intended deletion. In the resulting product, the start and stop codons of the corresponding gene separated by the *Nhe*I restriction site formed a markerless in-frame deletion. Similar approach was used to create chromosomal *BP2265*-triple FLAG tag in-frame fusion. The upstream and downstream fragments flanking the stop codon of the *BP2265* gene and carrying FLAG tag sequence at 3´and 5´end, respectively, were amplified and ligated via a *Psi*I site present in the FLAG sequence. In the resulting product, the FLAG tag was inserted in-frame in front of the TAG stop codon of the *BP2265* gene.

For both constructs, the final PCR product was ligated into the allelic exchange plasmid pSS4245 [37], the resulting plasmid was transformed into *E. coli* SM10 strain and transferred into *B. pertussis* B1917 by conjugation. After two recombination events, the strain carrying the desired mutation in *BP2265* was obtained and verified by sequencing of the amplified PCR product covering the adjacent chromosomal regions. The primers used in this study are listed in the Supplementary Table 2.

### RNA isolation, sequencing and data analysis

Cell pellets were suspended in TE buffer (10 mM Tris, 1 mM EDTA; pH 8.0) containing 1 mg/ml of lysozyme (Sigma) and total RNA was isolated from lysed cells using TRI Reagent (Sigma) according to manufacturer’s protocol. Removal of DNA was achieved by treatment of samples with TURBO DNA-free kit (Thermo Fisher Scientific). RNA quality and quantity was determined by agarose gel electrophoresis and using the Nanodrop One machine (Thermo Fisher Scientific). Furthermore, the RNA quality was assessed at sequencing facility (Institute of Advanced Biotechnology; https://www.iabio.eu/) on an Agilent 2100 Bioanalyzer device. All samples displayed RNA integrity numbers higher than 9.

Prior to sequencing, ribosomal RNA was depleted with NEBNext rRNA Depletion Kit (NEB). Libraries were prepared with NEBNext Ultra II Directional RNA Library Prep kit for Illumina (NEB) and sequenced on a NovaSeq 6000 platform (Illumina) using paired-end 101 base-pair read protocol at sequencing facility. RNA-seq data is deposited at the European Nucleotide Archive under project accession number PRJEB65304. Quality control of the obtained reads was done using FastQC (https://www.bioinformatics.babraham.ac.uk/projects/fastqc/). Quality trimming and adaptor removal was performed using Trimmomatic [38]. Next, reads were mapped to *B. pertussis* Tohama I transcriptome and quantified using Salmon algorithm [39]. Prior to differential expression (DE) analysis, unwanted variations caused by batch effects or library preparation were removed from the samples using the RUVs correction method of RUVseq (version 1.20.0) [40] in R (version 3.6.3). DE analysis was performed using DESeq2 [41], genes with a |log_2_ fold change| ≥ 1 and with adjusted *p*-value ≤ 0.05 were considered as significantly differentially expressed.

### LC-MS/MS and data analysis

Protein isolation, sample preparation and LC-MS/MS was performed as already described [13] (for details see Supplementary methods). The proteomics data were deposited to the ProteomeXchange Consortium via the PRIDE [42] partner repository with the dataset identifier PXD044487.

### Protein detection and immunoblotting

Samples of pelleted cells equivalent to 0.1 OD_600_ unit or samples of secreted proteins precipitated from culture supernatants equivalent to 1.0 OD_600_ unit were separated on 12.5% SDS-polyacrylamide gels and transferred onto a nitrocellulose membrane using Trans-Blot® Turbo™ system (Bio-Rad). Membranes were blocked with 5% skim milk and probed with in-house produced mouse polyclonal antibodies raised against recombinant proteins Bsp22, BopB, BopN, and BteA at a 1: 10 000 dilution followed by incubation with anti-mouse IgG antibodies conjugated with horse radish peroxidase (Cell Signalling Technology, Inc.) at a 1:10 000 dilution. The antibody–antigen complexes were visualized using SuperSignal West Femto chemiluminescent substrate (Thermo) according to standard protocol on G:Box Chemi XRQ device (Syngene).

### Co-immunoprecipitation

*B. pertussis* B1917 strain (control, non-tagged BP2265) and B1917 strain carrying FLAG-tagged BP2265 were grown in triplicates to mid-exponential phase (OD_600_ ≈ 1.5). Cultures (7 mL) were centrifuged (9,000 g, 15 min, 4 °C) and washed with 7 mL of TBS buffer (50 mM Tris, pH 7.5, 150 mM NaCl). Each pellet was then resuspended in 1.4 ml lysis buffer (TBS buffer, 1 mM EDTA, 0.1% IGEPAL CA-630, protease inhibitors) and sonicated in Protein LoBind® tubes (Eppendorf). The lysate was cleared by centrifugation (9,000 g, 10 min, 4 °C) and 1.2 ml of the lysate was mixed with pre-washed ANTI-FLAG M2 Magnetic Beads (15 µl packed gel volume; Sigma-Aldrich). Co-immunoprecipitation was performed for approximately 16 h at 4 °C and then the beads were washed three times in 600 µl TBS, divided into two parts and separated on magnetic rack. One part of the dry beads was immediately frozen at −80 °C (beads-bound fraction), and the second part of the beads was gently incubated three times in 75 µl elution buffer (100 mM glycine, pH 3) for 5 min at room temperature. Each elution was equilibrated with 7.5 µl of 10X TBS buffer, all three elutions were combined and frozen at −80 °C (elution fraction).

### Motility assay

Motility assays were performed according to two already described protocols. To follow the protocol of Hoffman and colleagues [43], *B. pertussis* and *B. bronchiseptica* RB50 cultures were grown in SSM overnight for 20 h, diluted to OD_600_ of 0.8 and 2 μl of the suspension was stabbed into SSM motility agar plates. Motility agar plates were prepared freshly before assay and consisted of 15 mL of SS medium containing 40 mM magnesium sulphate and 0.4% agar. Alternatively, motility was determined as described by Hiramatsu *et al*. [44]. *B. pertussis* and *B. bronchiseptica* cultures grown in SSM were washed, diluted to OD_600_ of 1.0 in Hanks’ balanced salt solution (Sigma-Aldrich) containing 20 mM Hepes (pH 7.4) and 0.1% BSA, and incubated at 37°C for 1 h. One microliter of bacterial suspensions was then stabbed into motility agar based on BGA (0.45% potato infusion powder, 0.55% NaCl, 1% casein hydrolysate, 1% glycerol, 1% BSA, and 0.4% agar). In both protocols, agar plates with *B. pertussis* and *B. bronchiseptica* strains were incubated in a humidified incubator at 37 °C for three days.

### Biofilm formation assay

*B. pertussis* cells grown on agar were suspended in SSM to a final OD_600_ of 0.3. Triplicate samples of diluted cultures (200 μl) were inoculated in parallel into 96-well non-treated tissue culture plates. The plates were incubated at 37 °C for up to three days in the absence or presence of 5% CO_2_. At each time point (24, 48, and 72 h), the loosly adherent and planctonic bacteria were discarded by washing the wells vigorously three times with water. Adherent cells were stained with 0.1% solution of crystal violet for 45 min. Plates were washed with water and dried, adsorbed crystal violet was then solubilized in 200 μl of 95% ethanol for 15 min. To quantify biofilm formation, 100 μl of stained solution was transferred to a microtitration plate and the *A*_595_ of the crystal violet staining was measured with a multi-well spectrophotometer Epoch (BioTek).

### Cell viability and cytotoxicity assays

Viability of infected THP-1 macrophages was determined spectrophotometrically as already described [15] using the WST-1 assay kit (Roche). THP-1 monocytes (ATCC; TIB-202) cultured in Dulbecco′s Modified Eagle′s Medium (DMEM, Sigma-Aldrich) were seeded in 48-well plates (2 × 10^5^ cells per well) and differentiated into macrophages as previously described [15]. The differentiated macrophages were infected in triplicates with *B. pertussis* cells diluted in 400 µl DMEM at a multiplicity of infection (MOI) of 50 bacteria per macrophage. After adddition of 40 µl of WST-1 substrate, the plates were centrifuged at 600 g for 3 min to facilitate interaction of bacteria with macrophages and incubated at 37 °C and 5% CO_2_. At indicated time points, the viability of infected and uninfected macrophages (treated in the same way and serving as controls, 100% viability) was determined as *A*_450_ of the formazan dye with a multi-well spectrophotometer Epoch (BioTek).

Cytotoxicity towards HeLa cells (ATCC; CCL-2) was determined essentially as already described for THP-1 macrophages [15] using CellTox^TM^ Green Cytotoxicity Assay (Promega). HeLa cells grown in DMEM without phenol red indicator were seeded in 96-well plate (1x 10^5^ cells per well), allowed to attach overnight at 37 °C and 5% CO_2_ and next day infected by adding 50 μl of a *B. pertussis* suspension (5x 10^6^ cells per well; MOI 50). After addition of 50 μl CellTox^TM^ reagent, plates were shortly centrifuged (400 g, 3 min) and then incubated in a Tecan Spark reader (37 °C, 5% CO_2_) for 10 h to measure the fluorescent signal.

## Results

### The putative T3SS chaperone BP2265 is required for the secretion of T3SS substrates and T3SS-mediated cytotoxicity

**Figure 1.**
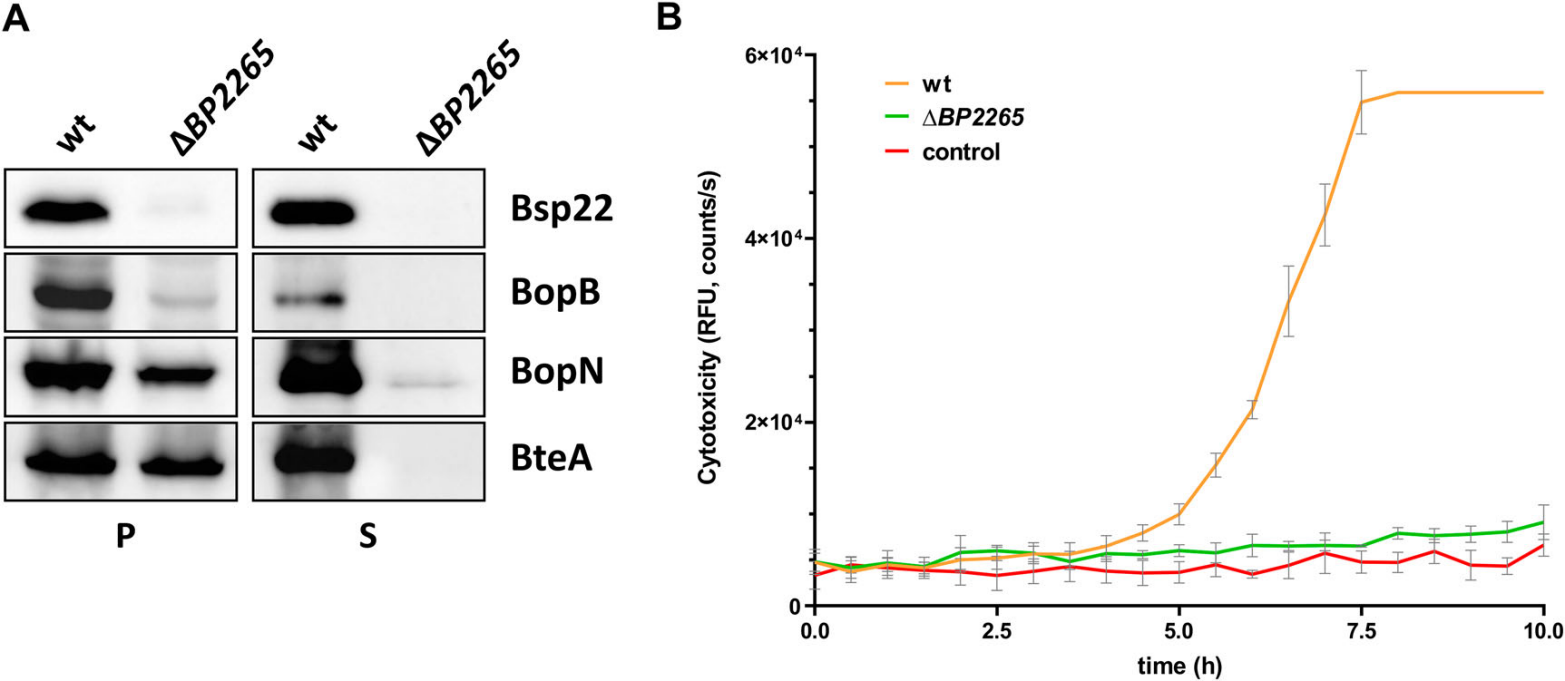
The Δ*BP2265* strain secretes strongly reduced amounts of T3SS substrates and is less cytotoxic compared to the wt strain. (A) Samples of pelleted cells (P) equivalent to 0.1 OD_600_ unit and precipitated supernatant proteins (S) equivalent to 1 OD_600_ unit were separated on 12.5% SDS-PAGE and analysed by immunoblotting using antibodies against Bsp22, BopB, BopN, and BteA proteins. Only the relevant parts of the membranes are shown. (B) Cytotoxicity towards HeLa cells was determined with the wt and Δ*BP2265* strain at MOI 50. Cells were infected in triplicates, uninfected cells served as controls. Immediately after addition of fluorescent dye, HeLa cells were incubated for 10 h (37 °C, 5% CO_2_) in the microplate reader. During incubation, the fluorescence of the DNA-binding dye CellTox Green, which is proportional to cytotoxicity, was measured every 30 min. The graph shows the mean values and the standard errors of the means. The result is representative of two independent experiments.

First, we analysed the homology between the BP2265 protein and the CesT chaperone of *E. coli*. While the sequence homology was rather low (18.2% identity, 29.3% similarity; see Supplementary Figure 1A), the structural homology between both proteins determined with the PyMOL tool was at 56% (Supplementary Figure 1B). To test the role of BP2265 in T3SS functionality, we first decided to construct a mutant carrying a markerless in-frame deletion of the *BP2265* gene in *B. pertussis* strain B1917 and test its ability to produce and secrete T3SS components. Strain B1917 was isolated from a patient with pertussis disease in the Netherlands in 2000 and is representative of current isolates in Europe [45]. In addition, for experimental tractability, it is beneficial to study T3SS function in *B. pertussis* in recent low-passage clinical isolates, as they exhibit greatly increased production and secretion of T3SS components compared with the high-passage reference strain Tohama I [6,7,9,18]. Western blot analysis revealed that compared to the wild-type (wt) strain, the strain lacking the *BP2265* gene produced comparable amounts of BteA and BopN, but strongly reduced amounts of Bsp22 and BopB proteins (Figure 1A). In addition, analysis of culture supernatants showed that the mutant strain secreted negligible amounts of all T3SS proteins tested. These results clearly demonstrated that the putative T3SS chaperone BP2265 is required for proper T3SS function in *B. pertussis*. Next, we reasoned that strongly decreased secretion of T3SS substrates, including the effector BteA, would result in reduced cytotoxicity of the mutant towards human HeLa cells, a model system sensitive to BteA activity [20,23]. Compared with the wt cells, infection with the Δ*BP2265* mutant yielded strongly reduced cytotoxicity to HeLa cells determined as changes in cell membrane integrity using the CellTox Green dye (Figure 1B).

### Transcriptomic profiling of the ΔBP2265 mutant

Given the structural homology of the BP2265 protein with the CesT family of chaperones and the complex role of CesT in regulating gene expression in *E. coli*, we hypothesized that the BP2265 protein also plays a broader role in the control of gene expression in *B. pertussis*. To test this hypothesis, we analysed and compared global transcriptomic and proteomic profiles between the *BP2265* mutant and the wt strain using the RNA-seq and LC-MS/MS methods, respectively.

**Figure 2.**
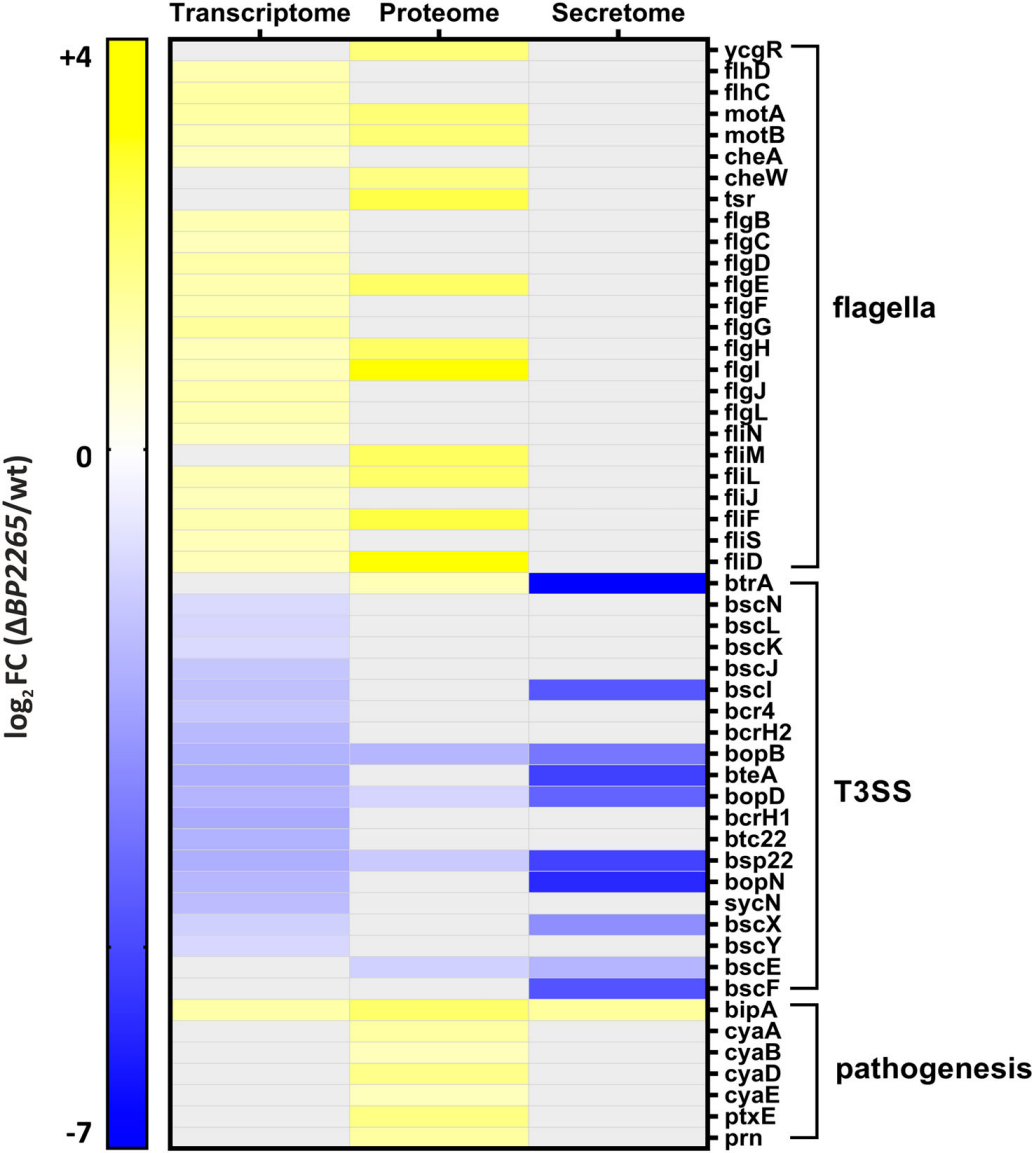
Heatmap showing flagellar, T3SS, and virulence genes displaying significantly altered omics profiles. The graph shows significant log_2_ fold changes (FC) in transcriptomic/proteomic profiles of genes/proteins that were downregulated (blue) or upregulated (yellow) in the Δ*BP2265* mutant compared to the wt strain. Transcriptome; gray filled cells indicate that the change in expression of the corresponding gene was not significant. Proteome and secretome; gray filled cells indicate that the change in abundance of the corresponding protein was not significant or the protein was not detected by mass spectrometry.

Previously, we showed that pre-incubation with blood affects the expression and secretion of T3SS components [13]. To make our study robust and minimize blood-specific effects, we used sheep blood from three different animals to obtain three biological replicates for RNA-seq analysis. On average, 10 million reads per sample were mapped to the *B. pertussis* genome. Principal component analysis revealed that despite some variability among replicates, samples of the wt strain cluster separately from samples of the mutant (Supplementary Figure 2A). Next, we performed differential expression (DE) analysis to identify the genes whose expression was affected by the deletion of the *BP2265* gene. The DE analysis revealed that a total of 54 genes were significantly modulated in the mutant (│log2FC│ ≥ 1; adjusted *p*-value < 0.05) (Supplementary Table 3). Among the 29 upregulated genes, we found 22 genes encoding flagellar proteins, two pseudogenes, two transcriptional regulator genes *BP0142* and *BP1496*, the autotransporter gene *BP0529*, one non-coding RNA gene, and the *bipA* gene encoding an outer membrane ligand-binding protein. Among the 25 downregulated genes, we found 20 genes belonging to the T3SS operon, two pseudogenes, one IS*481* transposase gene, and two non-coding RNA genes. These results indicated that the BP2265 regulon is highly specific, consisting primarily of T3SS and flagellar genes (Figure 2).

### Proteomic profiling of the ΔBP2265 mutant

We next tested whether the changes in gene expression profiles resulting from deletion of the *BP2265* gene translated into differential production and secretion of proteins. The cell-associated (bacterial pellets) and cell-free (filtered culture supernatants) fractions of wt and Δ*BP2265* strains cultures were analysed by LC-MS/MS. PCA performed with both datasets showed some degree of variability but the samples of the wt strain clustered apart of Δ*BP2265* samples (Supplementary Figure 2B,C). Label-free quantification of cell-associated proteins identified 221 proteins that showed significantly altered abundance (Supplementary Table 4). Interestingly, the vast majority of proteins (214 of 221; 96.8%) displayed increased abundance in the mutant. In good agreement with the transcriptomic data, we identified 7 flagellar and chemotaxis proteins and BipA protein among the 10 proteins with highest increase in quantity in the mutant (Figure 2). Of note, the amount of BtrA, adenylate cyclase toxin (ACT, also called CyaA) and components of its secretion system (CyaB, CyaD, CyaE), pertactin, and S5 subunit of the pertussis toxin was also significantly increased. Consistent with Western blot analysis and transcriptomic data, we found the T3SS translocon components BopB, BopD, and Bsp22 among the proteins with decreased levels (Figure 2).

Label-free quantification analysis of secreted proteins revealed that 47 proteins exhibited significant changes in quantity between the Δ*BP2265* mutant and the wt strain (Supplementary Table 5). Among the 19 proteins that showed reduced amounts in culture supernatants of the mutant, we identified 9 T3SS proteins, including BopN, BteA, Bsp22, BopB, BopD, and BscE, which were significantly reduced, and proteins BtrA, BscI, BscF, and BscX, which were not detected (Figure 2). Among the 28 proteins with increased abundance, we found predominantly putative periplasmic, membrane, and exported proteins including BipA.

### Immunoproteomics identifies anti-sigma factor BtrA as an interaction partner of CesT chaperone BP2265

In our next experiment, we applied immunoproteomics to identify the cognate substrate(s) of chaperone BP2265. B1917 cells producing non-tagged BP2265 protein (control) and FLAG-tagged BP2265 (serving as a “bait” protein) were grown to log phase in SSM, and cell lysates were incubated with anti-FLAG M2 magnetic beads. Western blot analysis of the proteins captured on the beads and proteins eluted from the beads revealed that a substantial portion of the BP2265 bait protein remains bound to the beads after elution (data not shown). Therefore, to obtain complete information on all possible interaction partners, we decided to analyse both fractions by the LC-MS/MS method. Both analyses yielded similar and highly specific results, as in addition to the bait protein BP2265, only the T3SS-secreted anti-sigma factor BtrA was highly enriched in both the captured and eluted (log_2_FC values > 5) protein fractions compared to non-tagged controls (Table 1). Among the significantly enriched proteins bound on the beads, we also found proteins BP1556, BP2173 and BP1894 to be approximately two-fold enriched. Analysis of the eluted proteins revealed only one additional significantly enriched protein, the sigma factor BtrS, which was most likely pulled down with its interaction partner BtrA. BtrS was also found among the proteins captured on the beads, but the enrichment did not reach the two-fold threshold (Table 1).

**Table 1.** Significantly enriched proteins identified by immunoproteomics.

Protein	Beads		Elution		
	log_2_FC^a^	q-value	log_2_FC	q-value	Annotation
BP2265	12.237	0	5.801	0	CesT family T3SS chaperone
BtrA	5.433	0	5.311	0	anti-ECF sigma factor BtrA
BtrS	*0*.*898*	0.015	1.891	0.003	ECF sigma factor
BP1556	1.154	0	ND		YrdC-like domain-containing protein
BP2173	1.121	0.008	ND		Putative ferredoxin
BP1894	1.072	0.001	ND		MarR-type domain-containing protein

^a^ Values that did not reach the two-fold threshold are in italics.

### In silico modelling of protein–protein interactions between BP2265 and BtrA

Our co-immunoprecipitation data suggested that the chaperone BP2265 interacts with BtrA. Therefore, we attempted to model this interaction using the deep learning system AlphaFold (AF) [46] which has recently been trained also for multimeric inputs. This model, named AF-Multimer (AF-M), has been shown to perform reasonably well in predicting interactions between two protein chains [47].

**Figure 3.**
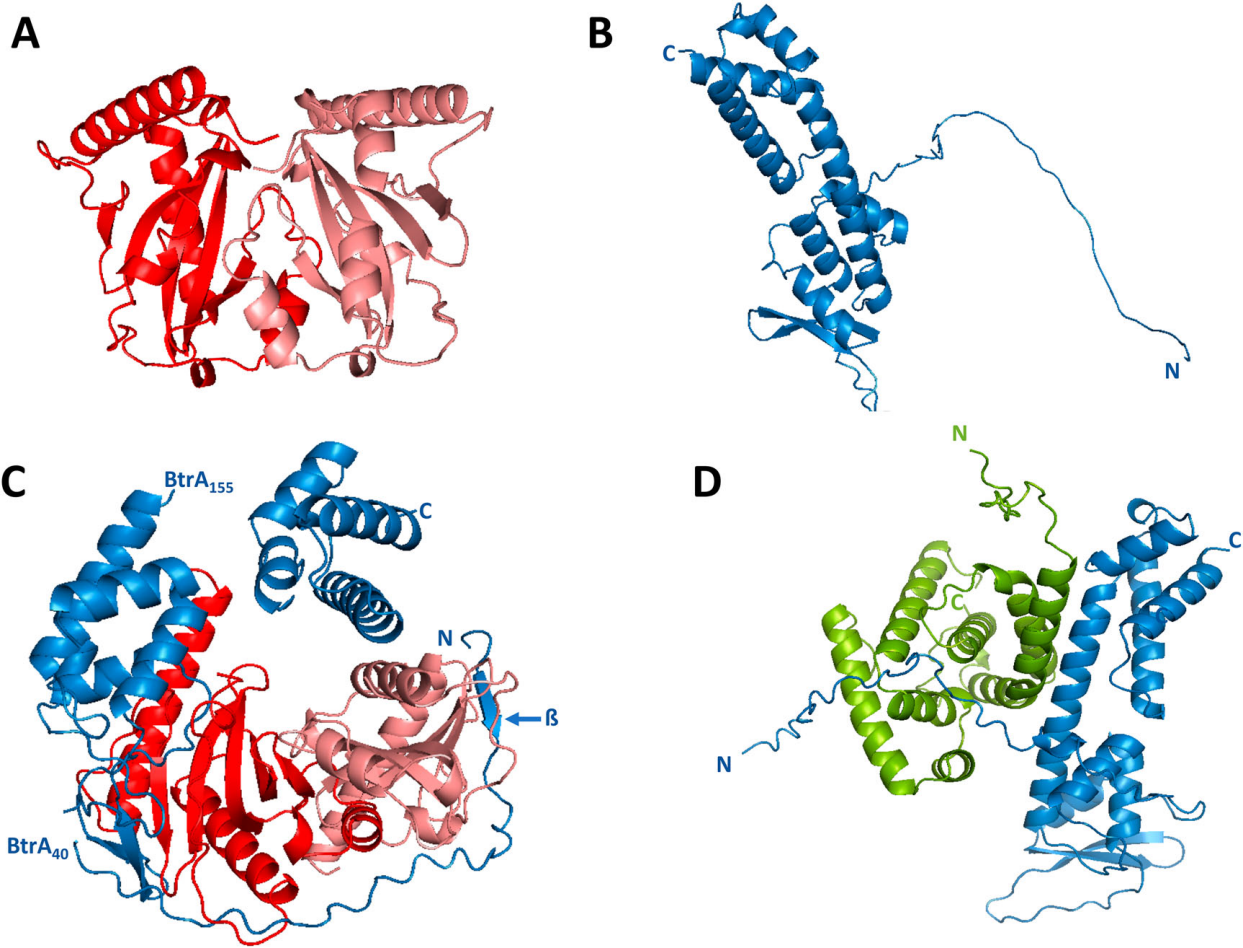
*In silico* modelling of T3SS proteins interactions. (A) Ribbon diagram of the dimer of BP2265 protein predicted by the AF-M algorithm. The monomers of BP2265 are shown in red and pink. (B) Ribbon diagram of the BtrA protein predicted by the AF system (Uniprot code Q7VWI6) with depicted N and C termini. (C) Ribbon diagram of the complex between the BP2265 dimer and the BtrA protein predicted using the AF-M system. Depicted are the N and C termini, the short N-terminal β-sheet, and the positions where the predicted BtrA structure is interrupted (residues 40 and 155). (D) Ribbon diagram of the BtrA-BtrS complex predicted by the AF-M system. The N and C termini of BtrA (blue) and BtrS (green) are depicted.

Similar to other chaperones, the CesT chaperone also functions as a dimer. Thus, we first used the AF-M system to model the BP2265 dimer yielding the typical heart-shaped structure (Figure 3A). Next, we used the predicted structures of the BP2265 dimer and the BtrA protein (Figure 3B) and modelled their interaction using AF-M program. The predicted structure suggests that the unstructured N-terminal region of BtrA (residues 1-40) is wrapped around the BP2265 dimer (Figure 3C). Furthermore, the predicted BtrA structure is interrupted at residues 40 and 155, indicating structural changes in BtrA caused by interaction with the BP2265 chaperone. Consequently, the central helix (residues 136-164) is split into two separate helices (residues 136–154 and residues 155-164). Interestingly, this model also suggests a small structural change in the outermost N-terminal part of BtrA, represented by the formation of a short β-sheet (residues 5-8). Because our co-IP experiments suggested a specific interaction between BtrA and BtrS proteins, we used the AF-M system to model this interaction (Figure 3D). The predicted heterodimer suggests an α-helix-mediated interaction between the central helix of BtrA (residues 136-164) and the N-terminal helix of BtrS (residues 21-38).

### Phenotypic characterization of the ΔBP2265 mutant

**Figure 4.**
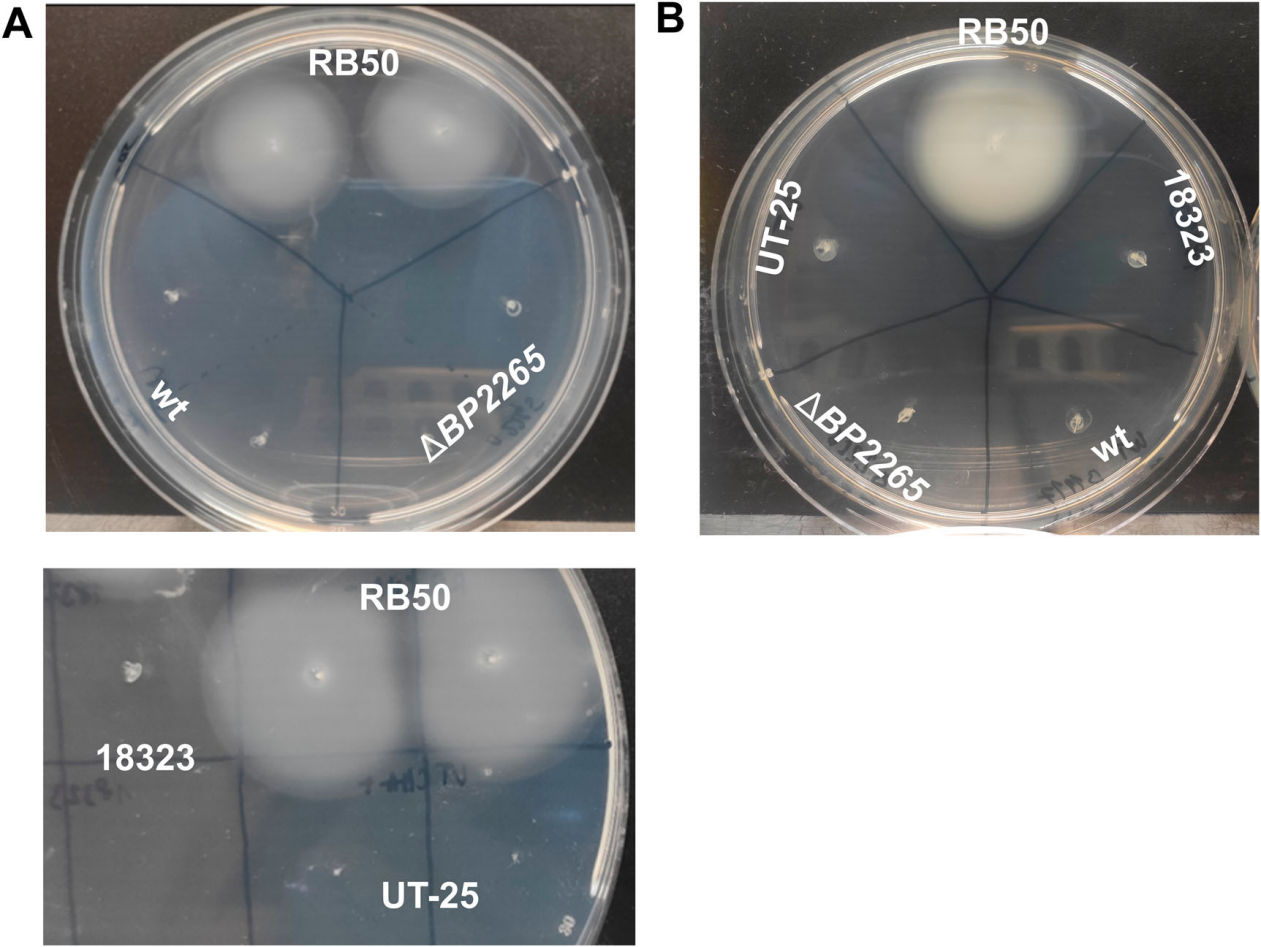
Motility assays. (A) Motility of *B. pertussis* strains B1917, its isogenic Δ*BP2265* mutant (upper panel), UT-25, and 18323 (lower panel) was tested together with *B. bronchiseptica* strain RB50 in 0.4% SSM motility agar containing 40 mM magnesium sulphate. (B) Motility of *B. pertussis* strains B1917, its isogenic Δ*BP2265* mutant, UT-25, and 18323 was tested together with *B. bronchiseptica* RB50 strain in 0.4% BGA-based motility agar. In all experiments, cultures of the tested strains were stabbed into a soft agar plate and incubated at 37 °C for three days. All images were taken after three days of incubation. The experiment was performed four times, and the figure shows the images of a representative experiment.

Next, we tested the possibility that the absence of the BP2265 chaperone leads, besides aberrant secretion of T3SS components, to additional phenotypic changes. The increased production of numerous flagellar proteins suggested that the mutant will exhibit increased motility. Therefore, we examined the motility of wt and Δ*BP2265* strains in soft SSM agar under modulatory conditions (40 mM magnesium sulphate), which have been shown to induce motility in *Bordetella* cells [43,48]. To verify that our experimental setup and conditions are permissive for studying motility, we also used the motile *B. bronchiseptica* RB50 strain. As shown in Figure 4A, unlike the control strain RB50, neither the mutant nor the wt strain were motile in our assay. Next, we tested two *B. pertussis* strains that have already been shown to be motile, namely the UT-25 isolate [43] and strain 18323 [44], however, also these *B. pertussis* strains were non-motile in contrast to *B. bronchiseptica* (Figure 4A). Finally, we tested the motility of all five strains according to another protocol [44]. Again, as shown in Figure 4B, only *B. bronchiseptica* strain RB50 exhibited motility on soft agar.

**Figure 5.**
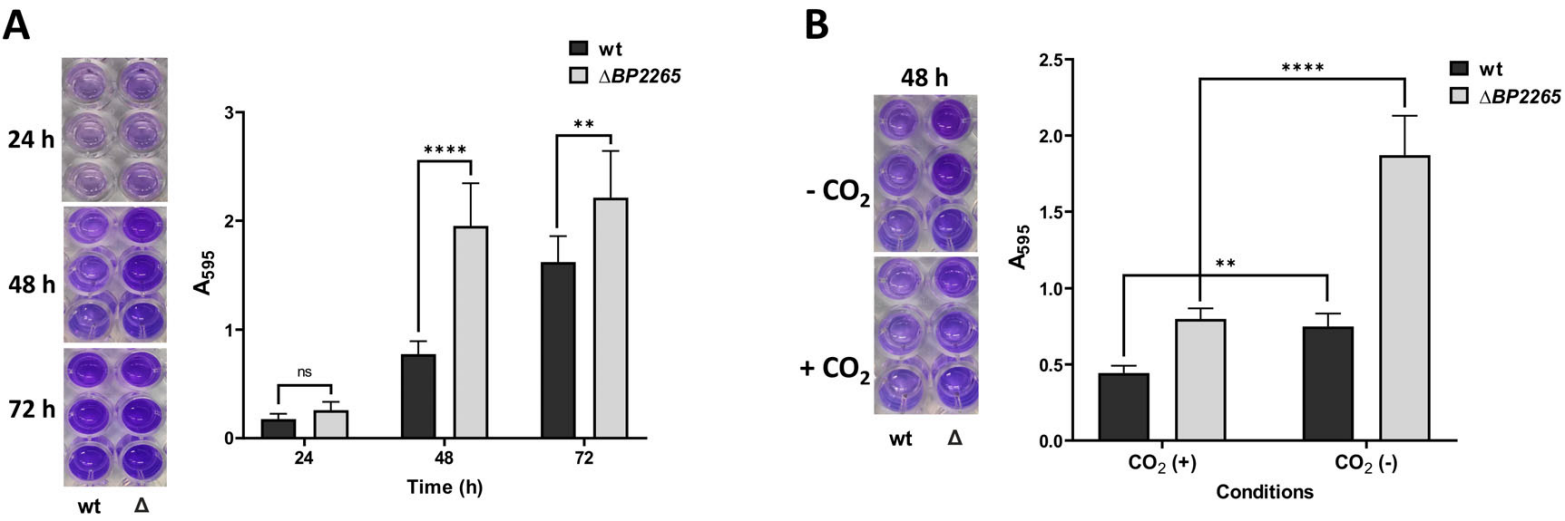
The Δ*BP2265* strain forms increased amounts of biofilm compared to the wt strain. (A) Biofilm formation was tested in triplicate samples of the B1917 strain (wt) and its isogenic Δ*BP2265* mutant (Δ) over three days under static aerobic conditions and determined by crystal violet staining. (B) Biofilm formation was determined in triplicate samples of the wt strain (wt) and the Δ*BP2265* mutant (Δ) after two days of incubation under static conditions in the absence (-CO_2_) or presence of 5% CO_2_ (+CO_2_) by crystal violet staining. Left panels; images of wells with crystal violet stain eluted from the biofilm formed by the corresponding strain. Right panels; graphs quantifying biofilm formation determined as *A*_595_ of crystal violet staining. Results are averages from three replicates, error bars represent standard deviations. Statistical analysis for panel A was performed using the two-way ANOVA test for multiple comparisons (Sidak´s test); ns, *p*-value > 0.05; **, *p*-value < 0.01; ****, *p*-value < 0.0001. Statistical analysis for panel B was performed using the two-way ANOVA test for multiple comparisons (Tukey´s test); **, *p*-value < 0.01; ****, *p*-value < 0.0001. Three independent experiments were performed for each panel, and one representative experiment is shown.

Thus, we focused on another aspect of the increased production of flagellar proteins, such as biofilm formation. Indeed, flagella have been shown to be required for the initial phase of biofilm formation in several bacteria, including *B. bronchiseptica* [49]. This observation prompted us to compare biofilm formation in the mutant and its parental strain. Both strains were incubated in SS medium without shaking, and biofilm production was monitored for several days using crystal violet method. While after one day of incubation both strains produced low but comparable amounts of biofilm, after two and three days the mutant produced significantly more biofilm than the wt strain (Figure 5A). Expression of flagellar and chemotaxis genes in *B. pertussis* has been shown to be reduced in the presence of 5% CO_2_ [11]. Compared to aerobic conditions, biofilm formation was significantly reduced in both wt and mutant strains in the presence of 5% CO_2_ (Figure 5B), providing another evidence that biofilm production requires flagella.

**Figure 6.**
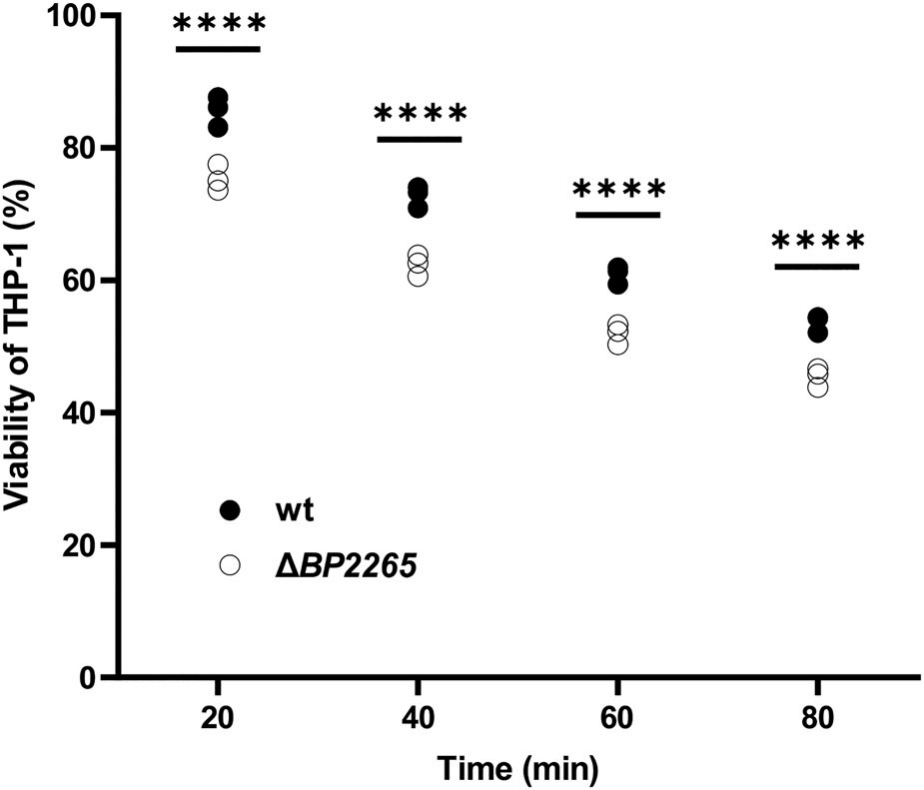
Viability of THP-1 macrophages infected with Δ*BP2265* mutant is reduced compared to the wt strain. THP-1 macrophages were infected in triplicate with the wt strain and the Δ*BP2265* mutant at MOI of 50 bacteria per macrophage. Infection was initiated by addition of WST-1 reagent and centrifugation of bacterial cells to facilitate the interaction with macrophages. During infection, *A*_450_ of samples, which is proportional to cell viability, was determined 20, 40, 60, and 80 min post-infection using multi-well spectrophotometer (Biotek). The absorbance of uninfected cells treated in the same manner was arbitrarily set to 100%. The dot plot shows the individual data points, and the labels above indicate statistical significance. Statistical analysis was performed using a two-way ANOVA test for multiple comparisons (Sidak´s test); ****, *p*-value < 0.0001. The result is representative of three independent experiments.

Finally, we examined whether increased production of virulence factors including adenylate cyclase toxin resulted in increased cytotoxicity of the mutant towards human macrophages. The viability of human monocyte-derived THP-1 macrophages infected with wt and Δ*BP2265* strains was determined spectrophotometrically using the cell proliferation reagent WST-1 at 20, 40, 60 and 80 min after infection. At all tested time points, the viability of macrophages infected with the mutant was significantly lower than that of macrophages infected with the wt strain (Figure 6).

## Discussion

In this work, we have focused on a relatively overlooked member of the T3SS locus of *B. pertussis*, the putative T3SS chaperone BP2265. Its high structural homology with *E. coli* CesT, the size of 15.9 kDa, the presence of an α helix in the C-terminal part, and the association with only one secreted substrate indicate that BP2265 represents a genuine class IA T3SS chaperone [50]. To elucidate the role of the BP2265 chaperone in *B. pertussis*, we combined omics techniques, immunoproteomics, standard biochemical methods, and deep learning system. First, we demonstrate that the Δ*BP2265* mutant shows reduced expression of most T3SS genes, secretes strongly reduced amounts of almost all T3SS substrates and, consequently, exhibits strongly reduced T3SS toxicity. Second, we show that BP2265 interacts specifically with the BtrA protein, and this interaction is supported by *in silico* data obtained with the AF-M program. BtrA cannot be *bona fide* considered a T3SS effector protein, and therefore BP2265 represents first non-flagellar T3SS chaperone required for the secretion of an anti-sigma factor.

Our immunoproteomic data revealed that the T3SS-specific sigma factor BtrS is co-purified with BtrA, confirming previous evidence for a specific interaction of these two proteins [29]. Given the role of BtrA in inhibiting the activity of the BtrS and the poor secretion of BtrA in the Δ*BP2265* strain, the reduced expression of most T3SS genes in the mutant was anticipated. On the other hand, the strongly reduced secretion but not production of early, intermediate, and late T3SS substrates is surprising and suggests that secretion of BtrA occurs as early as basal body formation. Our data demonstrate the pivotal role of the CesT chaperone BP2265 in BtrA secretion and indicate that its interaction with BtrA is a prerequisite for efficient assembly of the injectisome and efficient secretion of T3SS substrates. It appears that the role of BtrA in T3SS of *B. pertussis* is more complex and is not limited to silencing of BtrS-mediated transcription. In support, the *in silico* modelling analysis suggests that the BP2265 homodimer binds to the N-terminal portion of BtrA, consistent with both the proposed model [29] and previous data showing that the N-terminal part of BtrA is required for secretion [31]. Furthermore, predictions made by AF-M system indicate that BP2265 induces changes in the BtrA structure, including destabilization of the central helix, which in turn is predicted to interact with the N-terminal helix of BtrS. Thus, these observations suggest that the structural changes induced by BP2265 into the BtrA molecule prevent the interaction between BtrA and BtrS and promote secretion of BtrA. We propose to rename BP2265 as BtcB for the *Bordetella* type III chaperone of BtrA.

CesT plays a complex regulatory role in *E. coli* due to its interaction with the global regulator CsrA [34]. We did not identify an additional interaction partner for the chaperone BP2265, yet the BP2265 mutant exhibited altered expression profiles of numerous flagellar genes and several virulence genes. In the closely related *B. bronchiseptica*, the genes for flagella and chemotaxis are induced in the Δ*btrA* strain, indicating that BtrA inhibits expression of flagella, possibly by inactivating BtrS [29]. However, this is in contrast to a recent study showing that a *B. bronchiseptica* strain lacking BtrS exhibited increased expression of flagellar genes and higher motility compared to wt [51]. These results suggest that deletion of either the sigma factor or its antagonist may lead to similar expression pattern. The regulation of flagella expression in *Bordetella* is indeed intriguing and our data indicate that it is even more complex process, involving additional factors such as the CesT chaperone BP2265. Since our results showed that the Δ*BP2265* strain produces increased amounts of flagellar proteins, we assumed that the mutant would exhibit increased motility. Until recently, *B. pertussis* was considered non-motile bacterium, but two pieces of evidence showed that *B. pertussis* produces functional flagella and can be motile [43,44]. Unfortunately, we could not reproduce these results in our laboratory, although we used the same strains of *B. pertussis.* Furthermore, *B. bronchiseptica* assayed in parallel as a positive control was motile, indicating that our experimental conditions were favourable for motility testing.

Nevertheless, we demonstrate that increased production of several flagellar proteins in the mutant leads to increased biofilm production. Flagella are known to be implicated in biofilm formation as they are required for initial attachment to the surface [52]. Mutations introduced into flagellar genes resulted in reduced biofilm formation in several bacteria, including *B. bronchiseptica* [49, 53, 54]. In support, the production of biofilm was significantly increased in the Δ*BP2265* mutant compared to the wt strain. The presence of 5% CO_2_ significantly reduces the expression of flagellar genes in *B. bronchiseptica* [11] and therefore we speculated that under these conditions, flagella-dependent biofilm formation will be reduced as also shown in *Campylobacter jejuni* [55]. Our results are congruent with this assumption, as biofilm formation was reduced in the presence of 5% CO_2_. In addition to the flagellar proteins, the Δ*BP2265* mutant produced and secreted increased amounts of the intimin BipA. In pathogenic *E. coli* strains, intimins are required for the attachment to mammalian host cells [56]. Moreover, BipA is the major component of *Bordetella pertussis* biofilm [57] and therefore, we hypothesize that the increased production of BipA provides another basis for the increased biofilm formation by the mutant.

Finally, we show that the mutant producing moderately but significantly increased levels of several virulence factors, including ACT, exhibits increased cytotoxicity towards human macrophages compared with the wt strain. In *B. bronchiseptica*, the expression of adenylate cyclase toxin gene is activated by the BtrA [29]. Thus, it is conceivable that moderately increased levels of BtrA in the Δ*BP2265* mutant contribute to increased production of ACT and, consequently, increased cytotoxicity. Thus, our data suggest that the BtrA regulon in *B. pertussis* is larger and bears more similarity to *B. bronchiseptica* than previously thought.

In this work, we focused on the T3SS chaperone BP2265 of the CesT family and we demonstrate its requirement for the secretion of BtrA. We hypothesize that its chaperone activity towards the BtrA protein adds another layer in the regulatory circuit governed by the BtrA/BtrS node.

## Supplementary Material

Supplementary_methods_mass_spectroscopy

Supplementary_Table_5

Supplementary_Table_4

Supplementary_Table_3

Supplementary_Table_2

Supplementary_Table_1_revised

Supplementary_Figure_2

Supplementary_Figure_1
