# Supplementary material for: T3SS chaperone of the CesT family is required for secretion of the anti-sigma factor BtrA in Bordetella pertussis
Source: Emerg Microbes Infect. 2023 Nov 1;12(2):2272638. doi: 10.1080/22221751.2023.2272638 (PMC10732220; doi:10.1080/22221751.2023.2272638)
Supplement: Supplementary_methods_mass_spectroscopy [file TEMI_A_2272638_SM6449.docx]

**Protein isolation and sample preparation for proteomics**

Cell pellets were resuspended either in sample buffer for immunoblot analysis or in TEAB buffer (100 mM triethylammonium bicarbonate, pH 8.5, 2% sodium deoxycholate), lysed by sonication and centrifuged (9,000 g, 10 min, 4 °C) to clear the lysates. For analysis of secreted proteins, filtered supernatants of *B. pertussis* cultures were precipitated with 10% (w/v) trichloracetic acid (Sigma) overnight at 4 °C. Precipitated proteins were collected by centrifugation (14,000 g, 20 min, 4 °C), washed with 80% acetone and finally dissolved either in sample buffer for immunoblot analysis or in TEAB buffer. For LC-MS/MS analysis, protein concentrations were determined using BCA protein assay kit (Thermo Fischer Scientific) and 20 µg of protein per sample were used for analysis. Cysteines were reduced with 5 mM Tris(2-carboxyethyl)phosphine for 60 min at 60 °C and blocked with 10 mM methyl methanethiosulfonate (10 min, room temperature). Samples were digested with trypsin (trypsin to protein ratio 1:20) at 37 °C overnight. Digestion of samples was stopped by addition of trifluoracetic acid (Sigma) to a final concentration of 1% (v/v). Sodium deoxycholate was removed by extraction with ethylacetate and peptides were desalted on C18 column (Michrom Bio, Auburn, California, USA).

**LC-MS/MS and data analysis**

A Nano Reversed Phase Column (EASY-Spray Column, 50 cm × 75 μm internal diameter, PepMap C18, 2-μm particles, 100 Å pore size; Thermo Fisher Scientific) was used for liquid chromatography–mass spectrometry analysis. Mobile phase buffer A was composed of water and 0.1% formic acid. Mobile phase B was composed of acetonitrile and 0.1% formic acid. Samples were loaded onto the trap column (Acclaim PepMap300, C18, 5 μm, 300 Å wide pore, 300 μm × 5 mm; Thermo Fisher Scientific) at a flow rate of 15 μL/min. Loading buffer was composed of water, 2% acetonitrile, and 0.1% trifluoroacetic acid. Peptides were eluted with a gradient of phase B ranging from 4% to 35% over 60 min at a flow rate of 300 nL/min. Eluting peptide cations were converted to gas-phase ions by electrospray ionization and analyzed by Orbitrap Fusion (Thermo Fisher Scientific). Survey scans of peptide precursors from 350 m/z to 1,400 m/z were performed at 120 K resolution (at 200 m/z) with a 5 × 10^5^ ion count target.

Tandem mass spectrometry (MS2) was performed by isolation within a 1.5-Th window with the quadrupole, higher-energy collisional dissociation fragmentation with normalized collision energy of 30, and rapid scan mass spectrometry analysis in the ion trap. The MS2 ion count target value was set to 10^4^ and the maximal injection time was 35 ms. The precursors with charge state of 2–6 were sampled for MS2. The dynamic exclusion duration was set to 45 s with a 10 ppm tolerance around the selected precursor and its isotopes. Monoisotopic precursor selection was turned on. The instrument was run at top speed mode with cycles of 2 s.

Raw data were imported into MaxQuant software (version 1.5.3.8) [1] for label-free quantification of proteins. The false discovery rate (FDR) was set to 1% for peptides and minimum specific length of seven amino acids. The Andromeda search engine [2] was used for the MS/MS spectra search against the Uniprot *Bordetella pertussis* database (downloaded on November 2016). Protein abundance was calculated from obtained label-free protein intensities using the MaxLFQ algorithm [3]. For downstream analyses only proteins with more than four MS/MS spectral counts and which were detected in at least two of the three biological replicates were considered. In addition, the protein data set was reduced by potential and reverse contaminants and peptides identified only by site. Statistics and data interpretation were performed using Perseus 1.6.2.3 software [4]. Prior to data analysis, we used imputation method using sampling from normal distribution with parameters robustly estimated from analyzed data. Each abundance ratio was tested for significance with Student´s t-test, non-paired. The p-values were further adjusted for multiple testing correction to control the false discovery rate at cut off of 0.1 using the permutation test (number of randomization 250). Proteins with corrected p-value (q-value) < 0.1 were considered as significantly modulated.

1. Cox, J. and M. Mann, *MaxQuant enables high peptide identification rates, individualized p.p.b.-range mass accuracies and proteome-wide protein quantification.* Nat Biotechnol, 2008. **26**(12): p. 1367-72.

2. Cox, J., et al., *Andromeda: a peptide search engine integrated into the MaxQuant environment.* J Proteome Res, 2011. **10**(4): p. 1794-805.

3. Cox, J., et al., *Accurate proteome-wide label-free quantification by delayed normalization and maximal peptide ratio extraction, termed MaxLFQ.* Mol Cell Proteomics, 2014. **13**(9): p. 2513-26.

4. Tyanova, S., et al., *The Perseus computational platform for comprehensive analysis of (prote)omics data.* Nat Methods, 2016. **13**(9): p. 731-40.

5. Perez-Riverol, Y., et al., *The PRIDE database and related tools and resources in 2019: improving support for quantification data.* Nucleic Acids Res, 2019. **47**(D1): p. D442-D450.
