# Supplementary material for: T3SS chaperone of the CesT family is required for secretion of the anti-sigma factor BtrA in Bordetella pertussis
Source: Emerg Microbes Infect. 2023 Nov 1;12(2):2272638. doi: 10.1080/22221751.2023.2272638 (PMC10732220; doi:10.1080/22221751.2023.2272638)
Supplement: Supplementary_Table_5 [file TEMI_A_2272638_SM6447.pdf]

Supplementary Table 5. LC-MS/MS analysis of significantly* differentially secreted proteins

***(|log2FC| ≥ 1; adjusted p-value < 0.1) in Δ*BP2265* strain versus wt**

gene name	Gene ID	log_2FC	q-value	Protein IDs	Annotation
purM	BP0242	-2.57342	0.046167	Q7W0A7	Phosphoribosylformylglycinamidine cyclo-ligase
BP0301	BP0301	1.07811	0.059778	Q7W061	Putative ABC transporter substrate binding protein
dnaN	BP0490	-1.57563	0.042255	Q7VSE1	Beta sliding clamp
bteA	BP0500	-5.21671	0.064	Q7VSD3	Uncharacterized protein
BP0562	BP0562	1.06554	0.042615	Q7VS80	Putative exported protein
secB	BP0604	1.96138	0.041811	Q7VS46	Protein-export protein SecB
BP0735	BP0735	1.06127	0.076	Q7VZY4	Putative exported protein
BP0800	BP0800	-3.32242	0.096889	Q7VZT6	Probable zinc-binding dehydrogenase
ompA	BP0943	1.08838	0.049395	Q7VZG6	Outer membrane protein A
sbp	BP0966	1.46173	0.040127	Q7VZE6	Sulfate-binding protein
bipA	BP1112	1.51515	0.075385	Q7VZ27	Putative outer membrane ligand binding protein
BP1152	BP1152	1.19802	0.06125	Q7VYZ7	Putative exported protein
rimP	BP1245	1.07738	0.0415	Q7VYR4	Ribosome maturation factor RimP
livJ	BP1277	1.06116	0.045419	Q7VYN8	Leu/ile/val-binding protein
BP1364	BP1364	1.10455	0.040069	Q7VYH5	Putative amino-acid ABC transporter
BP1480	BP1480	1.07255	0.0602	Q7VY76	Putative exported protein
BP1485	BP1485	1.34195	0.05146	Q7VY72	Putative membrane protein
BP1532	BP1532	1.552	0.03939	Q7VY31	Putative amino acid-binding periplasmic protein
glnH	BP1573	1.56412	0.045225	Q7VXZ7	Glutamine-binding periplasmic protein
BP1675	BP1675	-1.12259	0.04432	Q7VXR3	Putative exported protein
BP1723	BP1723	3.14546	0.041412	Q7VXM8	Putative exported protein
tesA	BP1733	-1.71049	0.044593	Q7VXL8	Acyl-CoA thioesterase I
BP1850	BP1850	1.44288	0.037484	Q7VXC9	Putative exported protein
BP1887	BP1887	1.29618	0.054727	Q7VXB3	Putative exported protein
ppiB	BP1906	1.077	0.038098	Q7VX98	Peptidyl-prolyl cis-trans isomerase
btrA	BP2233	-7.01844	0.059	Q7VWI6	Uncharacterized protein
bscI	BP2249	-4.61781	0.072667	Q7VWI5	Putative type III secretion protein
bopB	BP2252	-3.80266	0.109	Q7VWI4	Putative outer protein B
bopD	BP2253	-4.3056	0.0688	Q79GQ3	Putative outer protein D
bsp22	BP2256	-5.16961	0	Q7VWI3	Putative secreted protein
bopN	BP2257	-5.79489	0.042667	Q79GQ0	Putative outer protein N
bscE	BP2263	-2.11224	0.052348	Q79GP4	Uncharacterized protein
bscF	BP2264	-4.71405	0.089091	Q79GP3	Putative type III secretion protein
BP2411	BP2411	-1.63084	0.098	Q7VW57	Uncharacterized protein
BP2418	BP2418	1.30086	0.046308	Q7VW51	Putative ABC transport protein
lolA	BP2472	1.07973	0.038733	Q7VW06	Outer-membrane lipoprotein carrier protein
BP2491	BP2491	2.72365	0.044	Q7VVY9	Putative cytochrome
BP2758	BP2758	1.15781	0.050571	Q7VVB9	Exported protein
BP2818	BP2818	1.30653	0.050167	Q7VV70	Lipoprotein
bfrG	BP2922	1.03274	0.044667	Q7VUZ3	Putative TonB-dependent receptor
tyrS	BP2954	-2.13203	0.057647	Q7VUW5	Tyrosine--tRNA ligase
BP2994	BP2994	1.19939	0.040772	Q7VUT0	Possible membrane protein
BP3037	BP3037	1.96748	0.048821	Q7VUP1	SWIB domain-containing protein
BP3218	BP3218	-2.78412	0.07	Q7VUA1	Putative membrane protein
BP3454	BP3454	-4.28422	0.046933	Q7VTR0	Putative lipoprotein
rpsG	BP3609	1.01604	0.057333	Q7VTD6	30S ribosomal protein S7
glmU	BP3730	-1.5507	0.048273	Q7VT27	Bifunctional protein GlmU
