# Supplementary material for: T3SS chaperone of the CesT family is required for secretion of the anti-sigma factor BtrA in Bordetella pertussis
Source: Emerg Microbes Infect. 2023 Nov 1;12(2):2272638. doi: 10.1080/22221751.2023.2272638 (PMC10732220; doi:10.1080/22221751.2023.2272638)
Supplement: Supplementary_Table_4 [file TEMI_A_2272638_SM6444.pdf]

## Supplementary Table 4. LC-MS/MS analysis of significantly* differentially produced proteins

**(|log2FC| ≥ 1; adjusted p-value < 0.05) in Δ*BP2265* strain versus wt**

| gene name | Gene ID | $log_2FC$ | q-value | Protein ID | Annotation |
|---|---|---|---|---|---|
| BP0506 | BP0506 | 4.16901 | 0.024 | Q7VSC7 | Uncharacterized protein |
| fliD | BP1410 | 4.1221 | 0.0184 | Q7VYD9 | Flagellar hook-associated protein 2 |
| fliF | BP1403 | 2.72206 | 0.027 | Q7VYE5 | Flagellar M-ring protein |
| flgI | BP1380 | 2.64044 | 0.01715 | Q7VYG2 | Flagellar P-ring protein |
| tsr | BP1030 | 2.63518 | 0.108 | Q7VZ95 | Methyl-accepting chemotaxis protein I |
| BP1528 | BP1528 | 2.51151 | 0.01932 | Q7VY35 | Threonylcarbamoyl-AMP synthase |
| cheW | BP1029 | 2.39659 | 0.036 | Q7VZ96 | Chemotaxis protein CheW |
| flgH | BP1379 | 2.3544 | 0.02335 | Q7VYG3 | Flagellar L-ring protein |
| fliL | BP1395 | 2.27043 | 0.036 | Q7VYF0 | Flagellar protein FliL |
| bipA | BP1112 | 2.24147 | 0.024 | Q7VZ27 | Putative outer membrane ligand binding protein |
| BP0314 | BP0314 | 2.13649 | 0.03284 | Q7W051 | Uncharacterized protein |
| rplL | BP0014 | 2.08513 | 0.03782 | Q7W0S0 | 50S ribosomal protein L7/L12 |
| motB | BP1025 | 2.08269 | 0.01964 | Q7VZ98 | Chemotaxis protein MotB |
| motA | BP1024 | 2.07961 | 0.01785 | Q7VZ99 | Chemotaxis protein MotA |
| ycgR | BP0877 | 2.05128 | 0.0195 | Q7VZM1 | Flagellar brake protein YcgR |
| BP2487 | BP2487 | 2.02927 | 0.027 | Q7VVZ3 | Uncharacterized protein |
| fliM | BP1394 | 1.95841 | 0.0216 | Q7VYF1 | Flagellar motor switch protein FliM |
| BP2867 | BP2867 | 1.92684 | 0.026 | Q7VV39 | Putative ABC transport proteins, ATP-binding component |
| ugpE | BP1283 | 1.8965 | 0.03631 | Q7VYN3 | glycerol-3-phosphate transport system permease protein |
| ptxE | BP3786 | 1.89377 | 0.0174 | P04981 | Pertussis toxin subunit 5 |
| phg | BP1767 | 1.85305 | 0.01357 | Q79GU5 | Autotransporter |
| mobA | BP1466 | 1.82206 | 0.04241 | Q7VY88 | Molybdenum cofactor guanylyltransferase |
| serA | BP0155 | 1.80861 | 0.03849 | Q7W0H7 | D-3-phosphoglycerate dehydrogenase |
| BP0699 | BP0699 | 1.7943 | 0.04567 | Q7W009 | Putative exported protein |
| cyaD | BP0762 | 1.78778 | 0.01156 | P0DKX9 | Protein CyaD |
| BP3052 | BP3052 | 1.77462 | 0.0144 | Q7VUN0 | Putative gamma-glutamyltranspeptidase |
| BP1088 | BP1088 | 1.77298 | 0.03838 | Q7VZ49 | UPF0761 membrane protein BP1088 |
| sdhC | BP2363 | 1.75706 | 0.02051 | Q7VW99 | Succinate dehydrogenase cytochrome B subunit |
| BP2705 | BP2705 | 1.75337 | 0.04058 | Q7VVG4 | Putative molybdenum-binding protein |
| BP1496 | BP1496 | 1.74492 | 0.03624 | Q7VY65 | Probable two-component response regulator |
| BP0625 | BP0625 | 1.7213 | 0.03306 | Q7VS28 | Probable acyl-CoA dehydrogenase |
| BP1437 | BP1437 | 1.703 | 0.02229 | Q7VYB2 | Probable class-I glutamine amidotransferase |
| BP2399 | BP2399 | 1.6753 | 0.01642 | Q7VW67 | Putative transcriptional regulator |
| BP3738 | BP3738 | 1.6435 | 0.0373 | Q7VT19 | SURF1-like protein |
| hslO | BP2379 | 1.59189 | 0.03226 | Q7VW86 | 33 kDa chaperonin |
| BP0634 | BP0634 | 1.55405 | 0.01984 | Q7VS22 | Probable acyl-CoA dehydrogenase |
| BP2144 | BP2144 | 1.54392 | 0.03717 | Q7VWQ7 | Uncharacterized protein |
| BP1428 | BP1428 | 1.53316 | 0.03848 | Q7VYC1 | Putative outer membrane protein |
| flgE | BP1376 | 1.51284 | 0.03395 | Q7VYG6 | Flagellar hook protein FlgE |
| prpC | BP2368 | 1.51057 | 0.02349 | Q7VW96 | Citrate synthase |
| BP0122 | BP0122 | 1.50843 | 0.03444 | Q7W0J4 | Sugar ABC transporter, ATP-binding protein |
| BP0876 | BP0876 | 1.49883 | 0.0176 | Q7VZM2 | Putative lipoprotein |
| BP3302 | BP3302 | 1.49769 | 0.0338 | Q7VU33 | Uncharacterized protein |
| sdhD | BP2362 | 1.49597 | 0.01775 | Q7VWA0 | Succinate dehydrogenase membrane anchor protein |
| BP0628 | BP0628 | 1.48324 | 0.054 | Q7VS26 | Putative pyruvate dehydrogenase E1 beta subunit |
| rpe | BP3266 | 1.47422 | 0.03638 | Q7VU62 | Ribulose-phosphate 3-epimerase |
| BP0641 | BP0641 | 1.47299 | 0.03358 | Q7VS15 | Putative exported protein |
| fabE | BP2997 | 1.4715 | 0.01842 | Q7VUS8 | Biotin carboxyl carrier protein of acetyl-CoA carboxylase |
| BP2792 | BP2792 | 1.46514 | 0.03284 | Q7VV93 | Uncharacterized protein |
| BP1920 | BP1920 | 1.463 | 0.02687 | Q7VX85 | Putative exported protein |
| BP2870 | BP2870 | 1.4508 | 0.01316 | Q7VV36 | Putative acid CoA ligase |

| | | | | | |
|---|---|---|---|---|---|
| prn | BP1054 | 1.43818 | 0.01076 | P14283 | Pertactin autotransporter |
| secE | BP0008 | 1.42689 | 0.02792 | Q7W0S5 | Protein translocase subunit SecE |
| BP0722 | BP0722 | 1.42237 | 0.01881 | Q7VZZ4 | Probable amidase |
| BP2418 | BP2418 | 1.3913 | 0.03946 | Q7VW51 | Putative ABC transport protein, substrate-binding component |
| BP3403 | BP3403 | 1.39062 | 0.03176 | Q7VTV0 | UTP--glucose-1-phosphate uridylyltransferase |
| cyaA | BP0760 | 1.38521 | 0.01687 | P0DKX7 | Bifunctional hemolysin/adenylate cyclase |
| fhaS | BP2667 | 1.38465 | 0.0104 | Q7VVJ2 | Adhesin |
| BP2341 | BP2341 | 1.38396 | 0.0208 | Q7VWB9 | Conserved hypothetical lipoprotein |
| BP2758 | BP2758 | 1.37923 | 0.04396 | Q7VVB9 | Exported protein |
| BP3750 | BP3750 | 1.36542 | 0.01733 | Q7VT07 | S-formylglutathione hydrolase |
| BP2452 | BP2452 | 1.36252 | 0.03694 | Q7VW21 | UPF0246 protein BP2452 |
| rnhB | BP1433 | 1.35921 | 0.01835 | Q7VYB6 | Ribonuclease HII |
| truA | BP1486 | 1.35811 | 0.03403 | Q7U363 | tRNA pseudouridine synthase A |
| asd | BP1484 | 1.35533 | 0.013 | P41399 | Aspartate-semialdehyde dehydrogenase |
| BP2921 | BP2921 | 1.35523 | 0.03381 | Q7VUZ4 | Putative exported protein |
| BP3425 | BP3425 | 1.35411 | 0.03546 | Q7VTT5 | Carbonic anhydrase |
| BP0635 | BP0635 | 1.33799 | 0.03259 | Q7VS21 | Probable enoyl-CoA hydratase |
| BP0627 | BP0627 | 1.32979 | 0.03328 | Q7VS27 | Probable enoyl-CoA hydratase/isomerase |
| cheB | BP1032 | 1.3103 | 0.0342 | Q7VZ94 | Protein-glutamate methylesterase/glutamine glutaminase |
| BP1445 | BP1445 | 1.30291 | 0.03642 | Q7VYA5 | Probable acyl-CoA dehydrogenase |
| tal | BP1451 | 1.30037 | 0.01606 | Q7VY99 | Transaldolase |
| potA | BP2347 | 1.29646 | 0.03309 | Q7VWB3 | Spermidine/putrescine import ATP-binding protein PotA |
| BP0455 | BP0455 | 1.28734 | 0.01486 | Q7VSG9 | Putative membrane protein |
| BP3058 | BP3058 | 1.28652 | 0.03615 | Q7VUM6 | Uncharacterized protein |
| uvrC | BP2081 | 1.2838 | 0.01667 | Q7VWV7 | UvrABC system protein C |
| lpxB | BP1432 | 1.28002 | 0.03566 | Q7VYB7 | Lipid-A-disaccharide synthase |
| purF | BP1415 | 1.27669 | 0.01889 | Q7VYD4 | Amidophosphoribosyltransferase |
| BP2403 | BP2403 | 1.25216 | 0.02446 | Q7VW64 | Electron transfer flavoprotein-ubiquinone oxidoreductase |
| nemA | BP1940 | 1.25017 | 0.03373 | Q7VX67 | N-ethylmaleimide reductase |
| pdhA | BP0629 | 1.2476 | 0.0216 | Q7VS25 | Putative pyruvate dehydrogenase E1 component |
| rpoP | BP0694 | 1.24364 | 0.03229 | Q7W012 | Nitrogen regulatory IIA protein |
| BP2071 | BP2071 | 1.2436 | 0.04008 | Q7VWW6 | Putative molybdenum-binding protein |
| BP0077 | BP0077 | 1.24276 | 0.04064 | Q7W0M5 | Putative exported protein |
| adhI | BP3751 | 1.24104 | 0.03411 | Q7VT06 | S-(hydroxymethyl)glutathione dehydrogenase |
| BP2671 | BP2671 | 1.22381 | 0.03305 | Q7VVI8 | Putative exported protein |
| BP0664 | BP0664 | 1.21694 | 0.01909 | Q7VRZ5 | Putative exported protein |
| BP1122 | BP1122 | 1.21495 | 0.01886 | Q7VZ20 | AsnC-family transcriptional regulator |
| BP2301 | BP2301 | 1.21387 | 0.04334 | Q7VWF6 | Putative amidohydrolase |
| relA | BP3587 | 1.21386 | 0.01748 | Q7VTF3 | Putative GTP pyrophosphokinase |
| BP3682 | BP3682 | 1.21085 | 0.03278 | Q7VT70 | Putative dehydrogenase |
| gpmB | BP1455 | 1.20122 | 0.018 | Q7VY96 | Probable phosphoglycerate mutase 2 |
| BP1360 | BP1360 | 1.18704 | 0.03546 | Q7VYH8 | 2-dehydropantoate 2-reductase |
| BP1707 | BP1707 | 1.18692 | 0.0229 | Q7VXP0 | Putative ABC-transport protein, ATP-binding component |
| bfrE | BP0857 | 1.18532 | 0.0193 | Q7VZP0 | Probable TonB-dependent receptor for iron transport |
| BP0624 | BP0624 | 1.18404 | 0.012 | Q7VS29 | Putative substrate-CoA ligase |
| putA | BP2749 | 1.18301 | 0.03203 | Q7VVC8 | Bifunctional protein PutA |
| BP2323 | BP2323 | 1.18287 | 0.03428 | Q7VWD7 | Conserved hypothetical membrane protein |
| acsA | BP2409 | 1.18145 | 0.03253 | Q7VW58 | Acetyl-coenzyme A synthetase |
| BP2770 | BP2770 | 1.18139 | 0.0385 | Q7VVA9 | Probable short-chain dehydrogenase |
| BP3001 | BP3001 | 1.17669 | 0.02207 | Q7VUS4 | Uncharacterized protein |
| BP2391 | BP2391 | 1.17487 | 0.01745 | Q7VW74 | Putative aminopeptidase |
| map | BP1418 | 1.17472 | 0.03411 | Q7VYD1 | Methionine aminopeptidase |
| minC | BP3227 | 1.17243 | 0.03694 | Q7VU93 | Probable septum site-determining protein MinC |
| ackA | BP1003 | 1.17112 | 0.0373 | Q7VZB6 | Acetate kinase |
| BP2333 | BP2333 | 1.16495 | 0.03291 | Q7VWC7 | Putative dioxygenase |
| BP2419 | BP2419 | 1.16466 | 0.01248 | Q7VW50 | Probable LysR-family transcriptional regulator |

| | | | | | |
|---|---|---|---|---|---|
| BP0251 | BP0251 | 1.15597 | 0.03712 | Q7W099 | LysR family transcriptional regulator |
| BP3438 | BP3438 | 1.15533 | 0.03623 | Q7VTS3 | IclR family transcriptional regulator |
| BP2377 | BP2377 | 1.14976 | 0.01946 | Q7VW88 | Putative AMP-binding protein |
| gltA | BP2358 | 1.14629 | 0.02024 | Q7VWA4 | Citrate synthase |
| hscA | BP1803 | 1.14611 | 0.03769 | Q7VXG7 | Chaperone protein HscA homolog |
| BP1359 | BP1359 | 1.14468 | 0.03425 | Q7VYH9 | Putative mandelate racemase |
| BP0752 | BP0752 | 1.14384 | 0.02128 | Q7VZX1 | LysR-family transcriptional regulator |
| BP1722 | BP1722 | 1.14179 | 0.03634 | Q7VXM9 | DNA_pol_B_exo2 domain-containing protein |
| ilvG | BP1860 | 1.14005 | 0.03839 | Q7VXC3 | Acetolactate synthase large subunit |
| BP3845 | BP3845 | 1.13737 | 0.02472 | Q7VST0 | Nitroreductase family protein |
| BP0665 | BP0665 | 1.13666 | 0.04227 | Q7VRZ4 | Thymidine diphosphoglucose 4,6-dehydratase |
| sdhA | BP2361 | 1.13453 | 0.02564 | Q7VWA1 | Succinate dehydrogenase flavoprotein subunit |
| BP2233 | BP2233 | 1.12952 | 0.02181 | Q7VWI6 | anti-ECF sigma factor BtrA |
| BP1092 | BP1092 | 1.12766 | 0.03306 | Q7VZ45 | Probable two-component histidine kinase |
| BP0208 | BP0208 | 1.12517 | 0.04568 | Q7W0D5 | Putative oxidoreductase |
| BP3400 | BP3400 | 1.12235 | 0.03955 | Q7VTV3 | SnoaL-like domain-containing protein |
| sodB | BP2761 | 1.12117 | 0.03787 | P37369 | Superoxide dismutase [Fe] |
| uvrB | BP1796 | 1.12095 | 0.02099 | Q7VXH4 | UvrABC system protein B |
| alaS | BP1836 | 1.11889 | 0.03395 | Q7VXE1 | Alanine--tRNA ligase |
| BP2521 | BP2521 | 1.11496 | 0.03579 | Q7VVW4 | Putative dehydrogenase |
| bioA | BP2165 | 1.11296 | 0.02 | Q7VWP0 | Adenosylmethionine-8-amino-7-oxononanoate transferase |
| hisH | BP3771 | 1.11022 | 0.04699 | Q7VSY8 | Imidazole glycerol phosphate synthase subunit HisH |
| BP3072 | BP3072 | 1.10892 | 0.03336 | Q7VUL4 | Putative acyltransferase |
| radA | BP1227 | 1.10569 | 0.03656 | Q7VYT2 | DNA repair protein RadA |
| queC | BP0214 | 1.10528 | 0.0371 | Q7W0D1 | 7-cyano-7-deazaguanine synthase |
| BP1874 | BP1874 | 1.10444 | 0.03811 | Q7VXB6 | Putative amidase |
| lep | BP2432 | 1.1006 | 0.01874 | Q79GN3 | Signal peptidase I |
| BP0992 | BP0992 | 1.09994 | 0.01659 | Q7VZC6 | Tw-component sensor kinase |
| rpsQ | BP3622 | 1.09979 | 0.04454 | Q7VTC4 | 30S ribosomal protein S17 |
| BP3190 | BP3190 | 1.09904 | 0.0335 | Q7VUB8 | TetR-family transcriptional regulator |
| BP2334 | BP2334 | 1.09843 | 0.0327 | Q7VWC6 | Putative ATP-dependent helicase |
| BP1865 | BP1865 | 1.09822 | 0.03385 | Q7VXB8 | GntR-family transcriptional regulator |
| tyrB | BP2858 | 1.09716 | 0.024 | Q7VV46 | Aromatic-amino-acid aminotransferase |
| BP3575 | BP3575 | 1.09671 | 0.03261 | Q7VTG5 | Putative exported protein |
| cyoA | BP2933 | 1.09464 | 0.02386 | Q7VUY2 | Ubiquinol oxidase subunit 2 |
| BP3528 | BP3528 | 1.09123 | 0.03231 | Q7VTK3 | Cys/Met metabolism PLP-dependent enzyme |
| BP0998 | BP0998 | 1.09075 | 0.04623 | Q7VZC1 | Ribosomal RNA small subunit methyltransferase E |
| glyQ | BP0033 | 1.0903 | 0.04012 | Q7W0Q6 | Glycine--tRNA ligase alpha subunit |
| BP0132 | BP0132 | 1.0897 | 0.03348 | Q7W0I8 | Putative membrane protein |
| BP2411 | BP2411 | 1.08951 | 0.04648 | Q7VW57 | Uncharacterized protein |
| cheD | BP3834 | 1.08656 | 0.01807 | Q7VST8 | Probable chemoreceptor glutamine deamidase CheD |
| BP1051 | BP1051 | 1.08596 | 0.01418 | Q7VZ80 | Probable D-alanyl-D-alanine carboxypeptidase |
| BP3320 | BP3320 | 1.08536 | 0.03383 | Q7VU20 | Uncharacterized protein |
| BP3857 | BP3857 | 1.0819 | 0.03857 | Q7VSS1 | Putative hydrolase |
| BP2317 | BP2317 | 1.07911 | 0.0331 | Q7VWE2 | Putative ribonuclease |
| glnD | BP1417 | 1.0786 | 0.03309 | Q7VYD2 | Bifunctional uridylyltransferase/uridylyl-removing enzyme |
| carB | BP1453 | 1.07551 | 0.0231 | Q7VY97 | Carbamoyl-phosphate synthase large chain |
| msbA | BP2321 | 1.0745 | 0.01812 | Q7VWD8 | Lipid A export ATP-binding/permease protein MsbA |
| purL | BP2668 | 1.07287 | 0.03392 | Q7VVJ1 | Phosphoribosylformylglycinamidine synthase |
| hisZ | BP2189 | 1.07027 | 0.03627 | Q7VWM0 | ATP phosphoribosyltransferase regulatory subunit |
| ileS | BP1753 | 1.06876 | 0.03751 | Q7VXK3 | Isoleucine--tRNA ligase |
| argA | BP2335 | 1.06438 | 0.03342 | Q7VWC5 | Amino-acid acetyltransferase |
| BP1590 | BP1590 | 1.06419 | 0.03762 | Q7VXY1 | Probable transcriptional regulator |
| BP3133 | BP3133 | 1.06235 | 0.0203 | Q7VUG3 | Putative LysR-family transcriptional regulator |
| cyaB | BP0761 | 1.06163 | 0.02081 | P0DKX5 | Cyclolysin secretion/processing ATP-binding protein CyaB |
| BP1446 | BP1446 | 1.061 | 0.03741 | Q7VYA4 | Enoly-CoA hydratase |

| | | | | | |
|---|---|---|---|---|---|
| prlC | BP0989 | 1.06024 | 0.03351 | Q7VZC9 | Oligopeptidase A |
| BP3506 | BP3506 | 1.06024 | 0.03252 | Q7VTL9 | Isochorismatase domain-containing protein |
| BP0697 | BP0697 | 1.05907 | 0.04724 | Q7W011 | ABC transporter |
| BP2364 | BP2364 | 1.05457 | 0.03977 | Q7VW98 | Putative GntR-family transcriptional regulator |
| BP1607 | BP1607 | 1.05362 | 0.03955 | Q7VXW7 | Probable LysR-family transcriptional regulator |
| BP0224 | BP0224 | 1.05256 | 0.03577 | Q7W0C1 | YCII domain-containing protein |
| cyaE | BP0763 | 1.05087 | 0.01236 | P0DKY0 | Protein CyaE |
| BP3136 | BP3136 | 1.04858 | 0.03501 | Q7VUG0 | Putative sulfatase |
| argS | BP0115 | 1.04664 | 0.02421 | Q7W0K0 | Arginine--tRNA ligase |
| BP0279 | BP0279 | 1.04607 | 0.03699 | Q7W078 | Uncharacterized protein |
| bfrD | BP0856 | 1.0444 | 0.03388 | P81549 | Probable TonB-dependent receptor BfrD |
| BP2727 | BP2727 | 1.04384 | 0.01632 | Q7VVE4 | SURF1-like protein |
| BP2815 | BP2815 | 1.03852 | 0.02829 | Q7VV73 | Uncharacterized protein |
| BP0048 | BP0048 | 1.03589 | 0.03681 | Q7W0P5 | Uncharacterized protein |
| pdxK | BP1321 | 1.03579 | 0.03678 | Q7VYK4 | Pyridoxine/pyridoxal/pyridoxamine kinase |
| clpA | BP2753 | 1.03508 | 0.03281 | Q7VVC4 | ATP-dependent clp protease ATP-binding |
| thrB | BP1084 | 1.03486 | 0.03699 | Q7VZ53 | Homoserine kinase |
| rpoA | BP3642 | 1.03409 | 0.04557 | P0A4E5 | DNA-directed RNA polymerase subunit alpha |
| BP2977 | BP2977 | 1.03406 | 0.02359 | Q7VUU6 | N-acetyltransferase domain-containing protein |
| BP3131 | BP3131 | 1.03371 | 0.03725 | Q7VUG5 | Putative oxygenase |
| rpsA | BP0950 | 1.03243 | 0.03637 | Q7VZG0 | 30S ribosomal protein S1 |
| BP3446 | BP3446 | 1.03182 | 0.03986 | Q7VTR6 | LysR family transcriptional regulator |
| BP3130 | BP3130 | 1.03171 | 0.03665 | Q7VUG6 | Putative cyclase |
| BP2397 | BP2397 | 1.02777 | 0.03251 | Q7VW69 | Putative ABC transporter ATP-binding subunit |
| tpm | BP0873 | 1.02719 | 0.03255 | Q7VZM5 | Thiopurine S-methyltransferase |
| pnp | BP0795 | 1.02703 | 0.02754 | Q7VZU0 | Polyribonucleotide nucleotidyltransferase |
| rpoB | BP0015 | 1.0267 | 0.03314 | Q7W0R9 | DNA-directed RNA polymerase subunit beta |
| dxs | BP2798 | 1.0258 | 0.03388 | Q7VV87 | 1-deoxy-D-xylulose-5-phosphate synthase |
| rkpK | BP3728 | 1.02556 | 0.03349 | Q7VT29 | UDP-glucose 6-dehydrogenase |
| ilvI | BP0789 | 1.0209 | 0.03963 | Q7VZU6 | Acetolactate synthase |
| BP2618 | BP2618 | 1.02011 | 0.03329 | Q7VVM9 | Putative transcriptional regulator (Fragment) |
| BP2298 | BP2298 | 1.01999 | 0.03688 | Q7VWF9 | Aminobutyrate aminotransferase |
| BP1218 | BP1218 | 1.01981 | 0.03678 | Q7VYT9 | Putative exported protein |
| BP2338 | BP2338 | 1.0197 | 0.0369 | Q7VWC2 | Putative transport protein |
| htpG | BP0074 | 1.01758 | 0.03825 | Q7W0M8 | Chaperone protein HtpG |
| fusA | BP3610 | 1.01753 | 0.03561 | Q7VTD5 | Elongation factor G |
| bplA | BP0093 | 1.01572 | 0.03798 | Q79H45 | Probable oxidoreductase |
| rimP | BP1245 | 1.01481 | 0.04387 | Q7VYR4 | Ribosome maturation factor RimP |
| moaE | BP2709 | 1.01304 | 0.02234 | Q7VVG0 | Molybdopterin converting factor |
| BP1756 | BP1756 | 1.01296 | 0.04324 | Q7VXK1 | SAM_MT_RSMB_NOP domain-containing protein |
| BP3744 | BP3744 | 1.00952 | 0.03731 | Q7VT13 | Cytochrome c oxidase subunit 2 |
| BP1900 | BP1900 | 1.00947 | 0.03655 | Q7VXA4 | Putative exported protein |
| BP3066 | BP3066 | 1.00876 | 0.03323 | Q7VUM0 | Methylenetetrahydrofolate reductase |
| mmsB | BP1447 | 1.00776 | 0.02158 | Q7VYA3 | 3-hydroxyisobutyrate dehydrogenase |
| BP2644 | BP2644 | 1.00718 | 0.03818 | Q7VVL1 | Putative glycerol-3-phosphate dehydrogenase |
| BP3435 | BP3435 | 1.00702 | 0.0464 | Q7VTS5 | Uncharacterized protein |
| mnmA | BP2893 | 1.00509 | 0.03562 | Q7U359 | tRNA-specific 2-thiouridylase MnmA |
| BP1467 | BP1467 | 1.00408 | 0.04337 | Q7VY87 | Iron-sulfur cluster carrier protein |
| BP3599 | BP3599 | 1.00328 | 0.02255 | Q7VTE3 | Uncharacterized protein |
| BP2378 | BP2378 | 1.00178 | 0.01114 | Q7VW87 | Putative transferase |
| ispF | BP0866 | 1.00057 | 0.04043 | Q7VZN1 | 2-C-methyl-D-erythritol 2,4-cyclodiphosphate synthase |
| alr | BP2228 | -1.04774 | 0.02411 | Q7VWJ0 | Alanine racemase, catabolic |
| bopD | BP2253 | -1.19821 | 0.01275 | Q79GQ3 | Type III secretion system translocon subunit BopD |
| bscE | BP2263 | -1.33649 | 0.04211 | Q79GP4 | T3SS chaperone |
| BP1189 | BP1189 | -1.37834 | 0.04557 | Q7VYW4 | Lipoprotein |
| bsp22 | BP2256 | -1.50383 | 0.0323 | Q7VWI3 | Type III secretion system needle tip complex Bsp22 |

| | | | | | |
|---|---|---|---|---|---|
| grpE | BP2501 | -1.96473 | 0.03435 | Q7VVY0 | Protein GrpE |
| bopB | BP2252 | -2.04111 | 0.03086 | Q7VWI4 | Type III secretion system translocon subunit BopB |
