# Supplementary material for: T3SS chaperone of the CesT family is required for secretion of the anti-sigma factor BtrA in Bordetella pertussis
Source: Emerg Microbes Infect. 2023 Nov 1;12(2):2272638. doi: 10.1080/22221751.2023.2272638 (PMC10732220; doi:10.1080/22221751.2023.2272638)
Supplement: Supplementary_Table_3 [file TEMI_A_2272638_SM6442.pdf]

Supplementary Table 3. Genes that were significantly* differentially expressed

***(|log2FC| ≥ 1; adjusted p-value < 0.05) in Δ*BP2265* strain versus wt**

gene	name	log_2FC Δ/wt	padj	annotation
Candidate_Transcript_278::as		-3.67703	3.47E-11	
BP2265	BP2265	-2.89968	1.81E-10	Uncharacterized protein
BP0041	BP0041	-2.6865	3.65E-08	Transposase
BP2254	bcrH1	-2.39739	6.81E-83	Putative regulatory protein
BP1652	BP1652	-2.3179	1.46E-20	pseudogene
BP0500	BP0500	-2.29452	6.17E-86	Uncharacterized protein
BP2256	bsp22	-2.26054	3.12E-61	Putative secreted protein
BP2252	bopB	-2.15595	8.18E-90	Putative outer protein B
BP2255	BP2255	-2.14983	2.95E-34	Uncharacterized protein
BP2253	bopD	-2.11332	6.4E-65	Putative outer protein D
BP2257	bopN	-2.00955	8.01E-37	Putative outer protein N
BP2251	bcrH2	-1.97437	2.05E-44	Putative regulatory protein
BP1610		-1.95728	1.34E-42	pseudogene
BP2258	BP2258	-1.88862	4.04E-13	Uncharacterized protein
BP2249	bscI	-1.77914	3.17E-29	Putative type III secretion protein
BP2228	alr	-1.68917	4.09E-22	Alanine racemase, catabolic
BP2229	BP2229	-1.67507	2.92E-25	Putative inner membrane transport protein
BP2248	bscJ	-1.60102	3.82E-34	Lipoprotein
BP2250	bcr4	-1.58858	1.67E-32	Uncharacterized protein
Candidate_Transcript_071::as		-1.4692	5.6E-08	
BP2259	BP2259	-1.34177	8.09E-05	Uncharacterized protein
BP2246	bscL	-1.17262	3.42E-12	Putative type III secretion protein
BP2260	BP2260	-1.1412	0.001975	Uncharacterized protein
BP2247	bscK	-1.07468	2.77E-05	Uncharacterized protein
BP2245	bscN	-1.06466	1.81E-10	Putative ATP synthase in type III secretion system
BP0529	BP0529	1.00085	7.09E-07	Autotransporter
BP1028	cheA	1.01922	5.45E-05	Chemotaxis protein CheA
BP1496	BP1496	1.02973	1.26E-05	Probable two-component response regulator
BP1393	fliN	1.03507	0.001222	Flagellar motor switch protein FliN
BP1399	fliJ	1.04651	0.011767	Flagellar protein FliJ
BP1374	flgC	1.06007	2.17E-06	Flagellar basal-body rod protein FlgC
BP1026		1.07052	3.77E-05	pseudogene
BP0142	BP0142	1.07294	0.001493	GntR family transcriptional regulator
BP1409	fliS	1.08488	0.006914	Flagellar secretion chaperone FliS
BP1379	flgH	1.08758	6.85E-07	Flagellar L-ring protein
BP1410	fliD	1.08992	4.86E-10	Flagellar hook-associated protein 2
BP1380	flgI	1.12064	9.89E-07	Flagellar P-ring protein
BP0176		1.12233	5.45E-05	pseudogene
BP1373	flgB	1.17114	3.93E-05	Flagellar basal body rod protein FlgB
BP1395	fliL	1.17559	0.001413	Flagellar protein FliL
BP1377	flgF	1.17585	1.81E-10	Flagellar basal body protein
BP1025	motB	1.19182	1.14E-05	Chemotaxis protein MotB
BP1383	flgL	1.20865	4.71E-08	Flagellar hook-associated protein 3
BP1403	fliF	1.22165	5.74E-06	Flagellar M-ring protein
BP1022	flhD	1.23613	2.28E-11	Flagellar transcriptional regulator FlhD S
BP1376	flgE	1.23937	1.95E-15	Flagellar hook protein FlgE
BP1381	flgJ	1.2835	2.57E-06	Peptidoglycan hydrolase
BP1375	flgD	1.31117	7.7E-13	Basal-body rod modification protein FlgD
BP1112	bipA	1.37057	2.22E-27	Putative outer membrane ligand binding protein
BP2627		1.38545	9.89E-23	pseudogene
BP1023	flhC	1.39236	4E-10	Flagellar transcriptional regulator FlhC
Candidate_Transcript_317::as		1.39549	2.02E-12	
BP1024	motA	1.39659	2.04E-11	Chemotaxis protein MotA
BP1378	flgG	1.53971	1.01E-23	Flagellar basal-body rod protein FlgG
