# Supplementary material for: T3SS chaperone of the CesT family is required for secretion of the anti-sigma factor BtrA in Bordetella pertussis
Source: Emerg Microbes Infect. 2023 Nov 1;12(2):2272638. doi: 10.1080/22221751.2023.2272638 (PMC10732220; doi:10.1080/22221751.2023.2272638)
Supplement: Supplementary_Table_2 [file TEMI_A_2272638_SM6441.docx]

**Supplementary Table 2. Primers used in this study**

| **Purpose of the primers** | **Sequence (5′-> 3′)*** |
| --- | --- |
| Construction of the *BP2265* mutant (upstream region) | F: ATAT*GAATTC*GGCGCGGAAAGACCAGCAG  R: TTA*GCTAGC***CAA**GGGAAACCATACGCTCGAAAT |
| Construction of the *BP2265* mutant (downstream region) | F: TTA*GCTAGC***TAG**GTCGGCGGCGACTGC  R: ATAT*GAATTC*CAGTATCGCCTTACCGCCATCG |
| Construction of chromosomal *BP2265-*triple FLAG tag fusion (upstream region) | F: ATAT*GAATTC*GGCGCGGAAAGACCAGCAG  R: TA*TTATAA*TCACCGTCATGGTCTTTGTAGTCGACGCCGCGCGTCAGC |
| Construction of chromosomal *BP2265-*triple FLAG tag fusion (downstream region) | F: TA*TTATAA*AGATCATGATATCGATTACAAGGATGATGATGACAAG**TAG**GTCGGCGGCGACTGC  R: ATAT*GAATTC*CAGTATCGCCTTACCGCCATCG |

*F, forward primer; R, reverse primer; sequences shown in italics indicate restriction enzyme recognition sites added for cloning purposes. The initiation (TTG) and stop (TAG) codons are shown in bold, the sequence corresponding to triple FLAG tag is underlined.
