# Supplementary material for: T3SS chaperone of the CesT family is required for secretion of the anti-sigma factor BtrA in Bordetella pertussis
Source: Emerg Microbes Infect. 2023 Nov 1;12(2):2272638. doi: 10.1080/22221751.2023.2272638 (PMC10732220; doi:10.1080/22221751.2023.2272638)
Supplement: Supplementary_Table_1_revised [file TEMI_A_2272638_SM6440.docx]

**Supplementary Table 1. Strains used in the study**

| **Strain** | **Description/application in the study** | **Reference** |
| --- | --- | --- |
| ***Escherichia coli*** |  |  |
| SM10(λ*pir*) | Maintenance and mobilization of pSS4245 vector/construction of mutants | [1] |
| XL1 blue | Cloning strain/construction of mutants |  |
| ***Bordetella pertussis*** |  |  |
| B1917 | Wild-type strain/WB, omics analyses, co-IP, motility, biofilm, cytotoxicity | [2] |
| Δ*BP2265* | B1917 carrying markerless deletion of *BP2265*/WB, omics analyses, motility, biofilm, cytotoxicity | This study |
| *BP2265-FLAG* | B1917 strain carrying in-frame *BP2265*-triple FLAG tag fusion/co-IP | This study |
| UT-25 | Wilde-type strain/motility | [3] |
| 18323 | Wilde-type strain/motility | ATCC |
| ***Bordetella bronchiseptica*** |  |  |
| RB50 | Wild-type strain/motility | [4] |

1. Simon, R., U. Priefer, and A. Pühler, *A Broad Host Range Mobilization System for In Vivo Genetic Engineering: Transposon Mutagenesis in Gram Negative Bacteria.* Biotechnology, 1983. **1** p. 784–791.

2. Bart, M.J., et al., *Complete Genome Sequences of Bordetella pertussis Isolates B1917 and B1920, Representing Two Predominant Global Lineages.* Genome Announc, 2014. **2**(6).

3. Brickman, T.J. and S.K. Armstrong, *The ornithine decarboxylase gene odc is required for alcaligin siderophore biosynthesis in Bordetella spp.: putrescine is a precursor of alcaligin.* J Bacteriol, 1996. **178**(1): p. 54-60.

4. Cotter, P.A. and J.F. Miller, *BvgAS-mediated signal transduction: analysis of phase-locked regulatory mutants of Bordetella bronchiseptica in a rabbit model.* Infect Immun, 1994. **62**(8): p. 3381-90.
