# Supplementary figures and images for: T3SS chaperone of the CesT family is required for secretion of the anti-sigma factor BtrA in Bordetella pertussis
Source: Emerg Microbes Infect. 2023 Nov 1;12(2):2272638. doi: 10.1080/22221751.2023.2272638 (PMC10732220; doi:10.1080/22221751.2023.2272638)

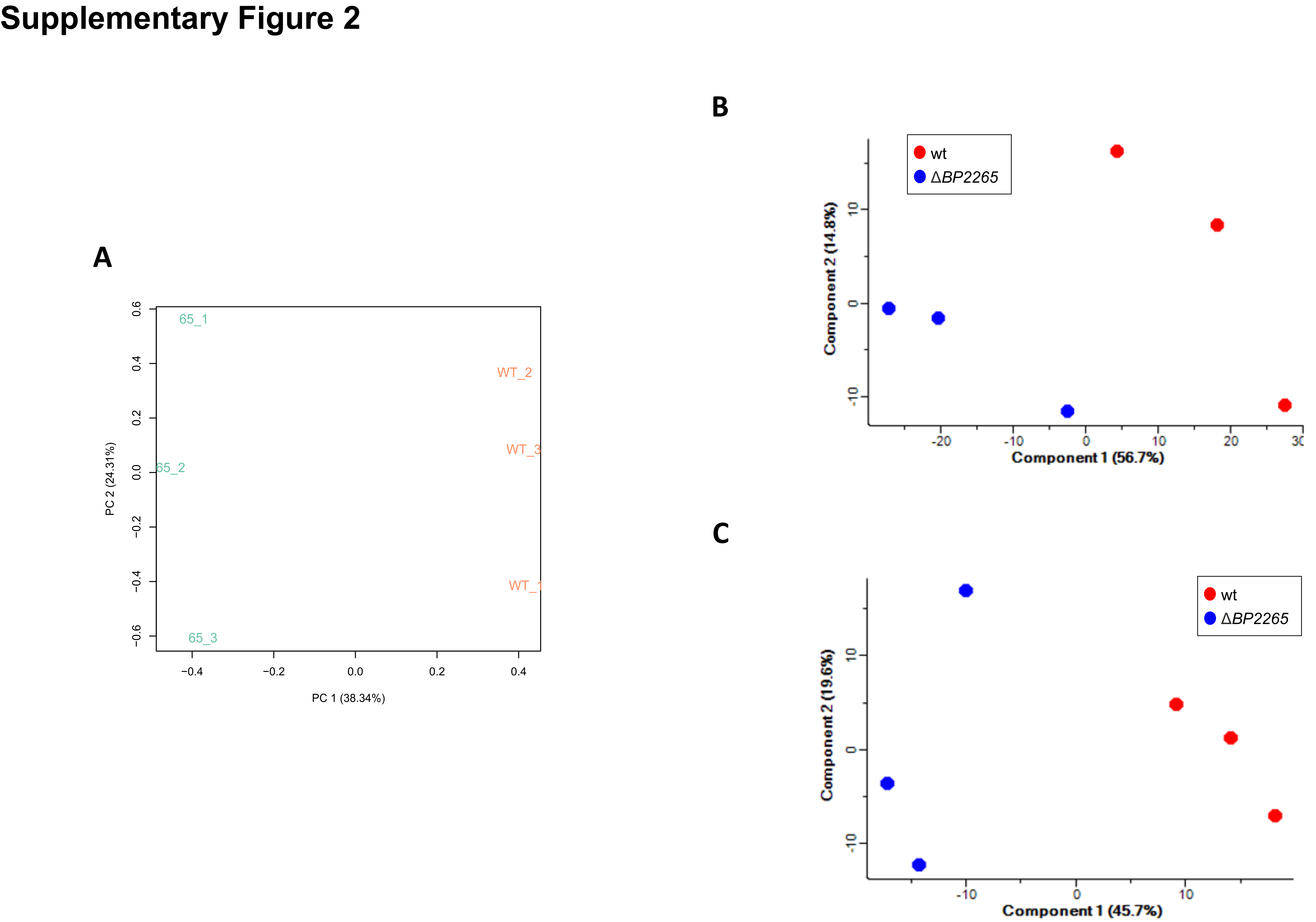

Supplement: Supplementary_Figure_2 [file TEMI_A_2272638_SM6438.tif]

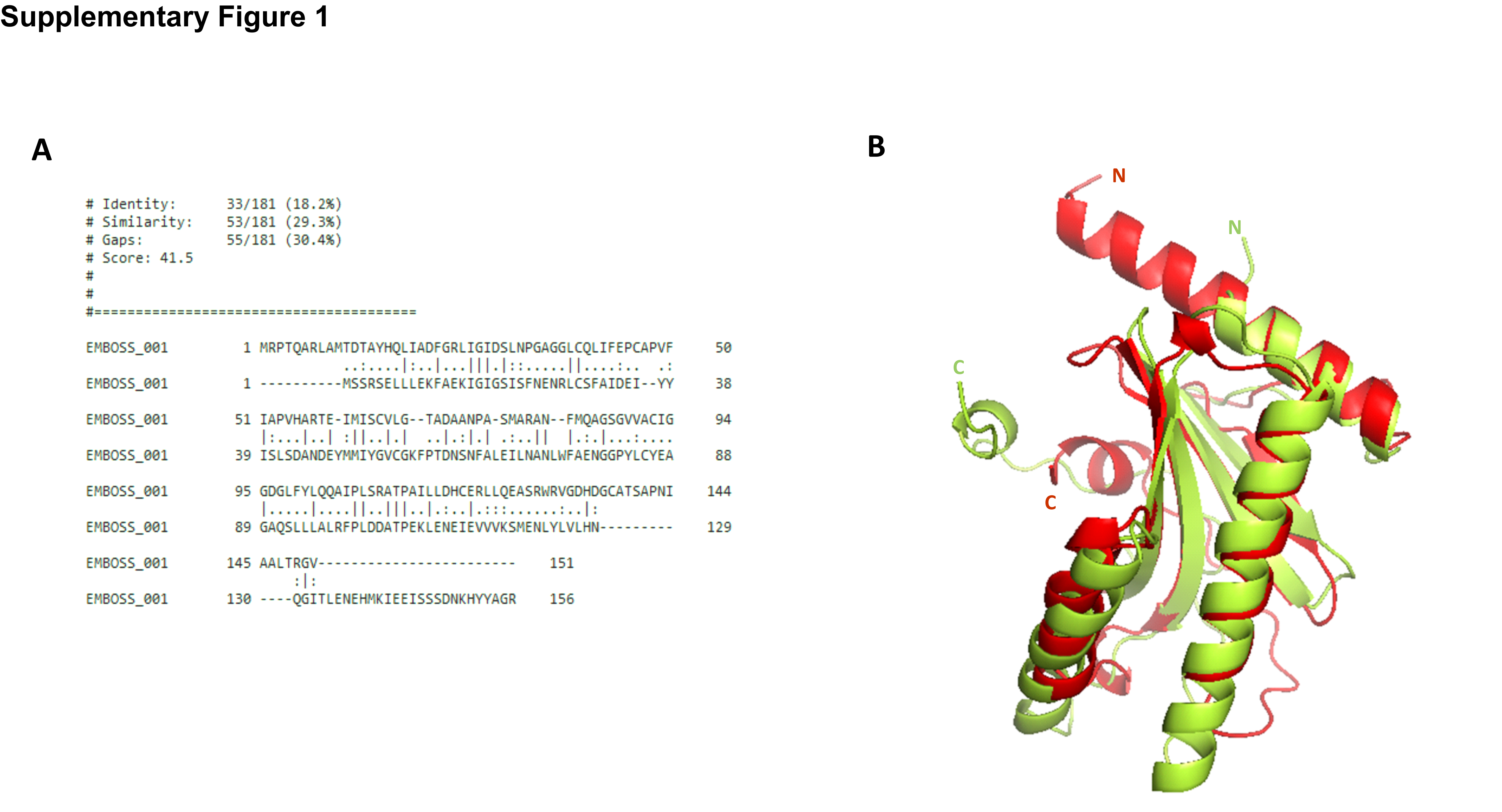

Supplement: Supplementary_Figure_1 [file TEMI_A_2272638_SM6435.tif]
